# Psychedelic Therapy's Transdiagnostic Effects: A Research Domain Criteria (RDoC) Perspective

**DOI:** 10.3389/fpsyt.2021.800072

**Published:** 2021-12-17

**Authors:** John R. Kelly, Claire M. Gillan, Jack Prenderville, Clare Kelly, Andrew Harkin, Gerard Clarke, Veronica O'Keane

**Affiliations:** ^1^Department of Psychiatry, Trinity College, Dublin, Ireland; ^2^Department of Psychiatry, Tallaght University Hospital, Dublin, Ireland; ^3^Trinity College Institute of Neuroscience, Trinity College, Dublin, Ireland; ^4^School of Psychology, Trinity College, Dublin, Ireland; ^5^Global Brain Health Institute, Trinity College, Dublin, Ireland; ^6^Transpharmation Ireland Ltd, Institute of Neuroscience, Trinity College, Dublin, Ireland; ^7^Discipline of Physiology, School of Medicine, Trinity College, Dublin, Ireland; ^8^School of Pharmacy and Pharmaceutical Sciences, Trinity College, Dublin, Ireland; ^9^Department of Psychiatry and Neurobehavioral Science, University College Cork, Cork, Ireland; ^10^APC Microbiome Ireland, University College Cork, Cork, Ireland

**Keywords:** psychedelics, hallucinogens, psilocybin, research domain criteria (RDoC), lysergic acid diethylamide (LSD), dimethyltryptamine (DMT), psychiatry

## Abstract

Accumulating clinical evidence shows that psychedelic therapy, by synergistically combining psychopharmacology and psychological support, offers a promising transdiagnostic treatment strategy for a range of disorders with restricted and/or maladaptive habitual patterns of emotion, cognition and behavior, notably, depression (MDD), treatment resistant depression (TRD) and addiction disorders, but perhaps also anxiety disorders, obsessive-compulsive disorder (OCD), Post-Traumatic Stress Disorder (PTSD) and eating disorders. Despite the emergent transdiagnostic evidence, the specific clinical dimensions that psychedelics are efficacious for, and associated underlying neurobiological pathways, remain to be well-characterized. To this end, this review focuses on pre-clinical and clinical evidence of the acute and sustained therapeutic potential of psychedelic therapy in the context of a transdiagnostic dimensional systems framework. Focusing on the Research Domain Criteria (RDoC) as a template, we will describe the multimodal mechanisms underlying the transdiagnostic therapeutic effects of psychedelic therapy, traversing molecular, cellular and network levels. These levels will be mapped to the RDoC constructs of negative and positive valence systems, arousal regulation, social processing, cognitive and sensorimotor systems. In summarizing this literature and framing it transdiagnostically, we hope we can assist the field in moving toward a mechanistic understanding of how psychedelics work for patients and eventually toward a precise-personalized psychedelic therapy paradigm.

## Introduction

Translational Psychedelic science is evolving rapidly (1–3). Initial data suggests that the dose dependent, transient, altered state of information processing induced by psychedelics can be harnessed by the psychotherapeutic process to lead to clinical benefits across a range of disorders. Accumulating preliminary clinical studies have shown that this synergistic combination of psychopharmacology and psychotherapy may improve outcomes in depression (4, 5), treatment resistant depression (TRD) (6–8) and addiction disorders (9, 10).

While results from ongoing well-powered double-blind randomized controlled trials (RCTs) will determine whether psychedelic therapy translates into clinical benefits for non-psychotic disorders in clinical psychiatry (11, 12), it has been proposed that psychedelic therapy may have broad therapeutic benefits *via* the attenuation of overly-restricted and maladaptive patterns of cognition and behavior (13, 14). Exploratory studies suggest potential benefits of psilocybin therapy in OCD (15), eating disorders (16) and migraine suppression (17), with ongoing RCTs of psilocybin therapy in MDD, bipolar disorder type II depression, alcohol use disorder, smoking cessation, cocaine addiction, opioid addiction, anorexia nervosa, depression in Mild Cognitive Impairment, OCD and various types of headaches (18).

A precise mechanistic understanding of psychedelics is challenging because of the synergistic action of pharmacotherapy and psychotherapy, together with the induction of a wide range of complex subjective experiences with marked individual variation (19). The primary initial pharmacological target of the classical psychedelics appears to be activation of 5-HT2A receptors (Box 1) particularly in cortical layer 5 pyramidal cells (20–27). A contemporary explanatory model—the Relaxed Beliefs under Psychedelics and the Anarchic Brain (REBUS)—proposes that psychedelics *via* action at 5-HT2A receptors in higher-order cortical regions (27) relax the typical constraints that higher order brain systems impose on emotions, cognitions, and sensory perceptions. This amounts to a decrease in the weight on (or precision of) prior beliefs, which in some disorders may be pathological (e.g., negative self-evaluations). This model proposes that psychedelics may facilitate an increase of information flow from bottom up signaling systems, opening the individual to information that they are otherwise biased to ignore or discount (13).

Box 1 **Classical psychedelics.**

**Table:** 

**Class**	**Primary receptor activation**	**Onset and duration of action**
**Indoleamines (aka tryptamines)**		
Psilocybin (phosphoryloxy-N,N- dimethyltryptamine) Psilocin (active metabolite of psilocybin, 4-hydroxy-DMT)	5-HT1, 5-HT2, 5-HT6 and 5-HT7 partial agonists	Onset 10–40 min po, peak 90–100 min, duration 4–6 h (most effects abate 6–8 h) Half-life: 2–3 h
N,N-dimethyltryptamine (DMT) 5-methoxy-DMT (5-MeO-DMT) Ayahuasca (aya) (DMT from *Psychotria viridis* plants and *Banisteriopsis caapi*, containing the potent MAO inhibitors beta-carboline alkaloids)	5-HT1, 5- HT2, 5-HT6, and 5-HT7 partial agonists	DMT IM onset within 2–5 min and can last 30–60 min DMT smoked or inhaled free-base <30 min DMT IV peak 5 min, abate by 30 min Aya: effects within 60 min, peak 90 min, can last 6 h
**Phenylalkylamines (synthetic “amphetamines”)**		
2,5-dimethoxy-4- iodoamphetamine (DOI) 2,5-dimethoxy-4- bromoamphetamine (DOB)	5-HT2A, 5-HT2B, 5-HT2C agonists	onset 1-2 h, duration 16–24 h
Mescaline		Peak within 2 h po, duration up to 8 h
**Semi-synthetic Ergolines**		
Lysergic acid diethylamide (LSD)	5-HT1, 5-HT2, 5-HT6 and 5-HT7 partial agonists D1 and D2 dopamine receptors and adrenergic receptors	po onset 30–45 min, peak 1–2.5 h, duration 9–12 h IV onset 3–5 min, peak 1 h, duration 9–10 h

The belief-recalibration process proposed by the REBUS model illustrates one mechanism through which psychedelic therapy may operate as a transdiagnostic therapeutic option for a broad range of disorders, particularly those with overly constrained beliefs or behaviors, such as major depression, anxiety and addiction disorders (13, 28). This model provides a framework for understanding their lack of efficacy in conditions such as psychosis spectrum disorders, where some have hypothesized there is insufficient constraint imposed on lower-level perceptions and cognitions. It follows that these disorders are exacerbated by psychedelics (29–31). Other overlapping models, focus on 5-HT2A receptor induced altered thalamic gating in cortico-striato-thalamo-cortical (CSTC) feedback loops (32–34).

As we accumulate more knowledge about the precise mechanisms of action, and how this might vary across individuals, we can begin to refine personalized treatment strategies. Currently available strategies to refine therapeutic outcomes include dose (and interval) optimization, modification of psychological interventions (perhaps dependent on the level of complexity or severity) and optimization of environmental ambiances/cues (setting) (35–38). Precise-personalized-predictive psychobiological markers are at an early stage of development, with exploratory clinical studies suggesting baseline Autonomic Nervous System activity (39), functional connectivity patterns (40–42) and cingulate cortical thickness (43), together with psychological factors such as absorption and openness (44–46) and language analysis (47) as potentially useful predictors of therapeutic outcomes. This research is reflective of a much broader advance toward individualized treatment approaches across all aspects of psychiatry, where the mantra is to move beyond one-size-fits-all toward more personalized care plans. In order to develop and build on these precision medicine approaches, there is growing consensus that research needs to traverse multiple levels of analysis.

**Figure 1:**
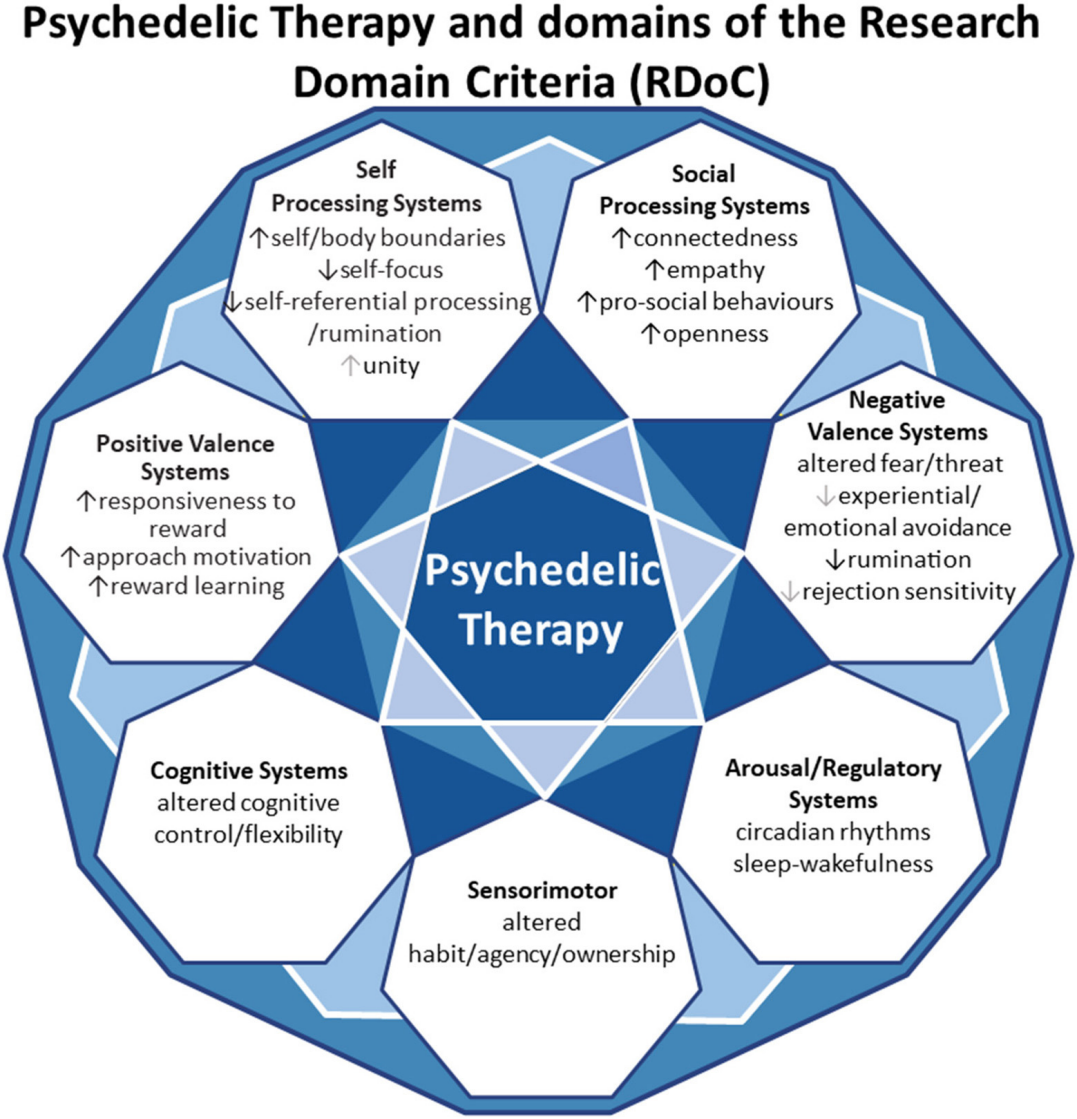
Transdiagnostic psychedelic therapy and domains of the research domain criteria (RDoC).

**Figure 2:**
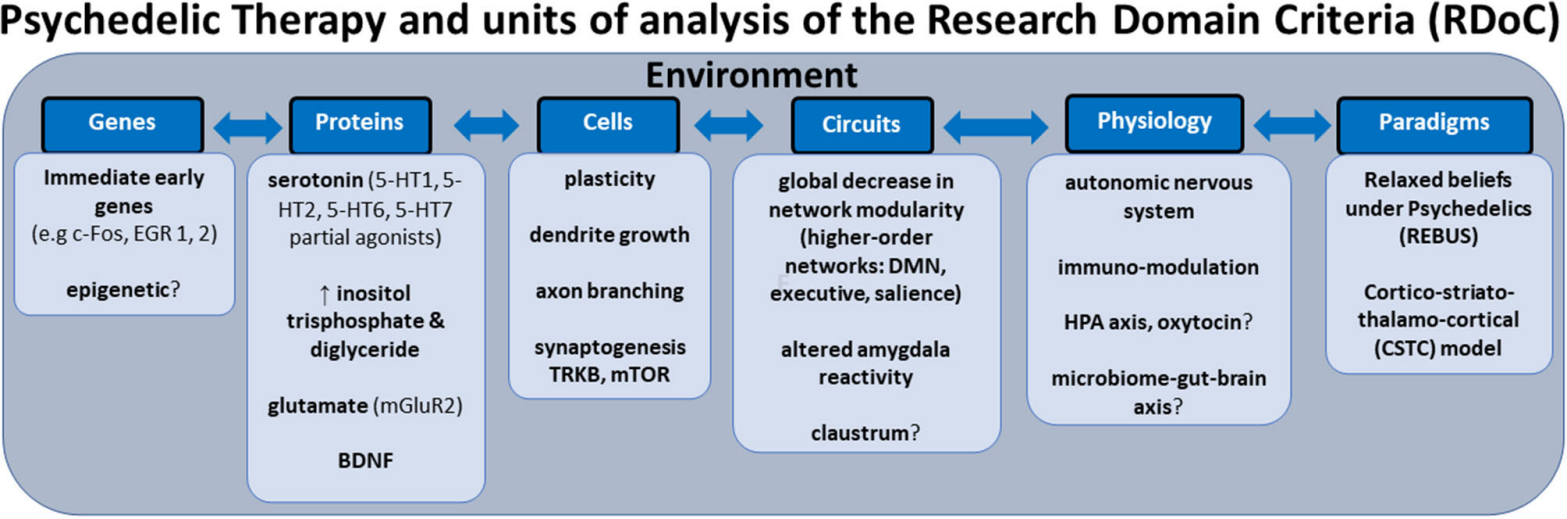
Transdiagnostic psychedelic therapy and units of analysis of the research domain criteria (RDoC).

In this review, we aim to anchor the accumulation of basic and applied research in psychedelics to the National Institute of Mental Health's Research Domain Criteria (RDoC), thereby adding structure to a fast-growing field. The transdiagnostic dimensional RDoC constructs are negative and positive valence systems, arousal regulation, social processing, cognitive and sensorimotor systems (Figure 1). In each section we will discuss, where available, research that spans multiple levels of analysis from genes, molecules, proteins, cells, circuits, physiology, behavior, self-report, and paradigms (Figure 2) (48–50). This review complements existing meta-analyses on the effects of psychedelic therapy (51–55) and recent reviews on the topic (18, 33). But in contrast, by framing and organizing the empirical data on psychedelics around the RDoC criteria, we aim to advance the field specifically toward a systems based precise-personalized psychedelic therapy paradigm.

## Integrating Psychedelic Therapy and the Research Domain Criteria

Personalized-precision psychiatry is impeded by two major issues that are partially related— (i) the reliance on categorical diagnostic systems and high levels of comorbidity and heterogeneity (56–60) and (ii) an over-reliance on small scale studies that cannot capture the complexity of mental health and illness, and as a result have failed to generate robust prediction/decision models needed for personalization. To the former point, there is broad consensus that categorical diagnostic labels, while necessary for pragmatic treatments in clinical settings, do not signify unitary, biologically credible, or informative markers of mental health and for example the overlap of previously presumed distinct psychiatric diagnoses, in terms of genes and brain networks, have been demonstrated by large neuroimaging (60–63) and genetic data sets (64–66). To the latter, there is increasing awareness that effect sizes in mental health science are generally small, regardless of whether variables are biological (67) or psychosocial (68). Thus, for personalization to occur, studies must move toward integrating multiple variables that have individually low predictive power—such approaches require large samples for accurate model development (69). Absent large datasets, a transdiagnostic and dimensional approach (compared to a categorical one) may do something to resolve both issues; if we can more accurately, validly and reliably capture mental health phenomena and the underlying biosignatures, then the effect sizes we observe will increase (59).

There are emerging signals that deconstructing categorical diagnoses into dimensional constructs may facilitate enhanced treatment precision. A recent clinical trial adopting an RDoC approach to the investigation of a selective κ-opioid receptor blocker for anhedonia across mood and anxiety disorders showed that this compound increased fMRI ventral striatum activation during reward anticipation compared to placebo (70). A study dividing MDD disorder symptoms into positive valence symptoms (impaired motivation, impaired energy, and anhedonia) and negative valence symptoms (anxiety and interpersonal sensitivity) showed that antidepressants were more effective for positive valence symptoms (71).

The evolving neuroscientific framework of the RDoC aims to integrate developmental processes and environmental inputs over the trajectory of the life course to determine the mechanisms underlying normal-range functioning and then how disruptions correspond to psychopathology. It is anticipated that the identification of targetable biosignatures that either cut across traditional disorder categories or that are unique to specific clinical phenomenon will improve outcomes for people with mental health disorders.

In the sections that follow, we will consider if and how psychedelic therapy operates across the RDoC domains in the hope that harnessing an integrative neuroscience systems model, encompassing environmental information exchange processes, may add the precision we need to transition to personalized psychedelic therapy practices that are transdiagnostic and evidence based. Although well-powered longitudinal clinical studies will be required to determine whether transdiagnostic dimensional biotypes or psycho-biotypes will optimize therapeutic response rates to psychedelic therapy (40, 41, 72), it is hoped that this review will lay a foundation for future research.

## Modulation of Negative Valence Systems

NVS are primarily responsible for responses to aversive (threat) situations or context, such as fear, anxiety, and loss (73, 74). Specifically, RDoC breaks NVS into acute threat (fear), potential threat (anxiety), sustained threat, loss and frustrative non-reward constructs. As we will outline in the next sections, psychedelic therapy may recalibrate NVS hyper-responsivity and positive valence systems (PVS) deficits across a range of psychiatric disorders.

### Loss

At the behavioral unit of analysis, the loss construct includes attentional biases to negative information, loss of motivation/drive, sadness, shame and rumination and is a component of several disorders but shares most features with depressive disorders (75). Some of the most important evidence for the operation of psychedelics on the NVS unsurprisingly comes from studies in depression. Pre-modern studies conducted during the 1950-60's first indicated a role of psychedelic therapy for depression and anxiety symptoms (76), which aligns with modern-era studies (77–79). The initial double-blind, randomized, placebo-controlled clinical studies in the modern-era of psychedelic therapy (psilocybin) showed an immediate and sustained antidepressant and anxiolytic effect in people with depressive symptoms associated with life-threatening cancer (80–82) (Table 1). In subgroups, these antidepressant and anxiolytic effects were sustained for several years (97), as were reductions in suicidal ideation and loss of meaning (98). Similarly, recent data suggest efficacy for another group with high levels of loss, those who survived Acquired immunodeficiency syndrome (AIDS) (101).

**Table 1:** Negative valence systems.

**Condition/Measures**	**Design**	**N, age**	**Psychedelic/dose**	**Clinical/neurobiological outcomes**	**References**
**Treatment-resistant depression (TRD)** MADRS, 5D-ASC, ASRS, EBI; EQ-5D-3L, GAD-7, HAM-D-17, MGH-ATRQ, MINI, MSI-BPD, PANAS, QIDS, QIDS-SR-16, SDS, STAR-C, STAR-P, WSAS	Randomized, double-blind	*n* = 233 94% no prior psilocybin experience	Psilocybin 1 mg (*n* = 79), or 10 mg (*n* = 75) or 25 mg (*n* = 79)	−6.6 points on change from baseline in MADRS total scores in 25 mg vs. the 1 mg dose at week 3 (*p* < 0.001) 25 mg group: 36.7% showed response at week 3, 29.1% were in remission at week 3, 24.1% were sustained responders at week 12 Serious treatment emergent adverse events: 6.3% in 25 mg, 8.0% in 10 mg, 1.3% in 1 mg. 12 patients reported suicidal behavior, intentional self-injury, and suicidal ideation (≥1 month post-psilocybin)	(8), unpublished
**Major depressive disorder (MDD)** QIDS-SR-16, BDI-1A, HAM-D-17, MADRS, FS, STAI, BEAQ, WSAS, SHAPS, WEMWBS, SIDAS, PRSexDQ, EBI, LEIS, PTCS	Double-blind, randomized, controlled	59 MDD (20F) 41 yrs (30 psilocybin, 29 escitalopram group)	Two psilocybin 25 mg po 3 weeks apart plus 6 weeks of daily placebo (psilocybin group) Or two psilocybin 1 mg 3 weeks apart plus 6 weeks of daily escitalopram po	No significant difference between groups in QIDS, mean (±SE) changes in the scores from baseline to week 6 were −8.0 ± 1.0 points in the psilocybin group and −6.0 ± 1.0 in escitalopram group Psilocybin decreased network modularity, or increased flexibility, of executive networks compared to the escitalopram group	(5, 42)
**MDD** GRID-HAMD, QIDS-SR, BDI, PHQ, C-SSRS, HAM-A, STAI	Randomized waitlist control trial (randomized immediately or after an 8-week delay) Antidepressant free	24 MDD (16F) 39.8 yrs (12.2)	Psilocybin (20 mg/70 kg and 30 mg/70 kg) Separated by 1.6 (mean) weeks	Significant decrease in GRID-HAMD and QIDS-SR scores at weeks 1 and 4 in the immediate treatment group compared to delayed treatment group Psilocybin increased cognitive flexibility for at least 4 weeks post-treatment (not correlated with antidepressant effects) Glutamate and N-acetylaspartate were decreased in the ACC at 1 week Greater increases in dFC between the ACC and PCC were associated with less improvement in cognitive flexibility Baseline dFC from the ACC predicted improvements in cognitive flexibility Greater baseline dFC was associated with better baseline cognitive flexibility but less improvement in cognitive flexibility	(4, 83)
**Treatment-resistant depression (TRD)** QIDS, BDI, STAI-T, SHAPS, MADRS, GAF, 11D-ASC, RRS BOLD fMRI Emotional Faces Images Task Cerebral blood flow (CBF)	Open label Antidepressant free	12 TRD (6F) 42.6 yrs (8 additional males at 6-month follow-up)	Psilocybin (10 and 25 mg 7 days later)	Significant reduction in depressive and anxiety symptoms and improvement in anhedonia scores from baseline to 1 week and 3 months 3-months: seven (58%) met criteria for response (BDI) 6-months: significant reductions in depression and anxiety symptoms (QIDS, BDI, STAI-T) Increased amygdala responses to emotional stimuli 1 day post-psilocybin, increased responses to fearful and happy faces in the right amygdala post-treatment. Right amygdala increases to fearful vs. neutral faces were predictive of clinical improvements at 1-week Decreases in CBF in the temporal cortex, including the amygdala (decreased amygdala CBF correlated with reduced depressive symptoms) Increase in FC between the amygdala and vPFC to occipital-parietal cortices during face processing Decreased vPFC-right amygdala FC in response to fearful and neutral (but not happy) faces associated with levels of rumination at 1 week (RRS) Emotional face recognition faster at follow-up in TRD but not controls and significantly correlated with a reduction in anhedonia Reduction of depressive symptoms at 5 weeks associated with high scores of acutely experienced pleasurable self-dissolution and by low scores for dread of ego dissolution Qualitative; change from disconnection to connection, and from emotional avoidance to acceptance	(6, 7, 40, 84–88)
**TRD** MADRS, HAM-D, MEQ30, BPRS+, CADSS, HRS at baseline, Day 1 (D1), D2 and D7 after dosing Serum BDNF and cortisol at D0 and D2	Randomized placebo-controlled trial Antidepressant free	29 TRD Aya = 14 (11F) 39.71 yrs (±1.26) Placebo = 15 (10F) 44.2 yrs (±11.98) 45 HCs (25F) 31.56 yrs (±9.90)	Ayahuasca 0.36 ± 0.01 mg/ml of N, N-DMT (mean ± S.D)	Significant reduction in depressive symptoms (MADRS) at D1, D2, and D7 vs. placebo Response rates significantly higher in the aya group at D7 (64 vs. 27%) Aya increased BDNF at D2 vs. placebo in both HCs and TRD but no significant differences between HC and MDD No significant differences in suicidality between aya vs. placebo Aya acutely increased salivary cortisol levels in both TRD and in HCs. 48 h after aya no difference in the cortisol awakening response between TRD and HCs Aya reduced CRP levels in both TRD (higher at baseline) and HCs compared to placebo TRD group treated with aya showed a significant correlation between larger reductions of CRP and lower depressive symptoms 48 h after aya. No significant changes in IL-6 levels	(89–93)
**MDD** SPECT (8 h post-dose) MADRS, HAM-D, BPRS (Anxious-Depression subscale) YMRS, CADSS Scales at 10 min before (baseline), 40, 80, 140, 180 min post-dosing and 1, 7, 14, 21 days	Open label Antidepressant free	17 MDD (14F) (3: mild, 13:moderate, 1:severe) 42.71 yrs (12.11)	Ayahuasca (2.2 mL/kg)	Significant decrease in MADRS and HAM-D (and subscales of the BPRS) across all time points No significant changes in YMRS scores Significant increases in the CADSS from 40 to 80 min Increased blood perfusion in the left nucleus accumbens, right insula and left subgenual area Significant acute (40, 80, 140, 180 min) and post-acute (1, 7, 14, 21 days) decreases in suicidality in secondary analysis using MADRS subscale among participants with baseline suicidality (*n* = 15)	(94, 95)
**MDD** HAM-D, MADRS, BPRS (Anxious-Depression subscale), YMRS Scales at 10 min before (baseline), 40, 80, 140, 180 min post-dosing and 1, 7, 14, 21 days	Open label Antidepressant free	6 MDD (4F) (2:mild, 3:moderate, 1:severe) 44.16 yrs (±13.55)	Ayahuasca (0.8 mg/mL DMT)	HAMD: significant decrease at D1, D7, D21 vs. baseline MADRS: significant decrease at 180 min, D1, D7, D21 vs. baseline BPRS-AD subscales: decrease at 140, 180 min, D1, D7, D14, D21 vs. baseline No significant changes in YMRS scores	(96)
**Depression and anxiety symptoms** in cancer GRID-HAM-D, HAM-A, BDI, STAI, POMS, HRS, 5D-ASC, PEQ MEQ30, M scale, BSI, MQOL, LAP-R, LOT-R, PIL, DTS, PEQ, FACIT-Sp, SROS, FMS	Randomized, double-blind, cross-over trial, counterbalanced	51 (25F) 56.3 yrs (1.4)	Psilocybin (1 or 3 mg/70 kg) and high dose (22 or 30 mg/70 kg) 5 weeks apart	Significant antidepressant and anxiolytic effects (HAMA, GRID-HAM-D) At 6 months; 83% (HAM-A) and 79% (GRID-HAM-D) met the criteria for response Significant improvements in BDI, STAI-state scale (STAI-S), STAI-T and POMS Mystical-type psilocybin experience on session day mediated therapeutic effect of psilocybin	(81)
**Adjustment disorder** and/or **generalized anxiety** in cancer HADS, BDI, STAI-S and STAI-T, BDI Outcomes assessed prior to crossover at 7 weeks, and up to 26 weeks after dosing session 2	Double-blind, placebo-controlled, crossover	29 (18F) 56.28 yrs (12.93)	Psilocybin (0.3 mg/kg) Or niacin (250 mg)	Immediate and sustained reductions in anxiety and depression symptoms (HADS, BDI, STAI-S and STAI-T) that remained significant until final follow-up. At 6.5-months: anti-depressant (BDI) or anxiolytic response rates (HAD-A) 60–80% At 4.5 yrs follow-up (16 alive, 15 participated); ~60–80% met criteria for clinically significant antidepressant or anxiolytic responses 71–100% attributed positive life changes to the psilocybin-assisted therapy and rated it among the most personally meaningful and spiritually significant experiences of their lives Reductions in suicidal ideation and loss of meaning	(82, 97, 98)
**Anxiety symptoms** in cancer patients EORTC-QLQ-30, STAI, HADS, Visual Analog Pain Scale, SCL-90-R Outcomes at baseline, 1-week, 2-months, 12-months	Double-blind, randomized, active placebo-controlled pilot, then into open-label crossover	12 (4F) 51.7 yrs	LSD (200 mcg) (*n* = 8) Or 20 mcg with an open-label crossover to 200 mcg after initial blinded treatment (*n* = 4) 2–3 weeks apart	2-months: significant reductions in STAI, sustained at 12 months Qualitative follow up at 12-months: insightful, cathartic, and interpersonal experiences, accompanied by a reduction in anxiety (77.8%), increase in quality of life (66.7%)	(99, 100)
**Anxiety/adjustment disorder** in advanced stage cancer 5D-ASC, STAI, BDI, POMS regularly up to 6 months	Double-blind placebo-controlled cross-over trial	12 (11F) 36–58 yrs (range)	Psilocybin (0.2 mg/kg) or niacin (250 mg) 2 separate dosing sessions	Significant decreases were observed in STAI scores at 3-months follow-up, and BDI scores at 6-months All 12 participants completed the 3-month follow-up 8 completed the 6-month follow-up (two subjects died and two became too ill to continue)	(80)
**Obsessive compulsive disorder (OCD)** YBOCS, VAL at 0, 4, 8, and 24 h, HRS at 8 h	Open label proof-of-concept pilot Antidepressant free (failed to respond to at least 1 SSRI for 12 weeks)	9 (2F) 40.9 yrs (±13.2)	Psilocybin po (25, 100, 200, and 300 mcg/kg at 1-week intervals)	23–100% decrease in YBOCS score (no dose response)	(15)

*PHQ, Patient Health Questionnaire; QIDS, Quick Inventory of Depressive Symptoms; SHAPS, Snaith-Hamilton Pleasure Scale; STAI, The State-Trait Anxiety Inventory (STAI) trait scale (STAI-T); POMS, Profile of Mood States; HAM-A, Hamilton Anxiety Rating Scale; GRID-HAM-D; HADS, Hospital Anxiety and Depression Scale; POMS, Profile of Mood States; HAM-D, Hamilton Rating Scale for Depression; MADRS, Montgomery-Asberg Depression Rating Scale; BPRS, Brief Psychiatric Rating Scale; YMRS, Young Mania Rating Scale; BHS, Beck hopelessness scale; SPECT, single photon emission tomography; CADSS, Clinician Administered Dissociative States Scale; DASS, Depression, Anxiety, and Stress Scale; DPES, Dispositional Positive Emotion Scale; PANAS-X, Positive and Negative Affect Schedule - X; 5D-ASC, 5-Dimensions Altered States of Consciousness questionnaire; CADSS, Clinician Administered Dissociative States Scale; SPECT, single photon emission tomography; HRS, Hallucinogenic Rating Scale; MEQ, Mystical Experience Questionnaire; F, female; HC, healthy controls; FC, functional connectivity; C-SSRS, Columbia-suicide severity rating scale; PFC, prefrontal cortex; vPFC, ventromedial prefrontal cortex; GAF, Global Assessment of Functioning; aya, ayahuasca; BDNF, Brain-derived neurotrophic factor; TRD, treatment-resistant depression; DMT, Dimethyltryptamine; YBOCS, Yale-Brown Obsessive Compulsive Scale; HRS, Hallucinogen Rating Scale; BPD, borderline personality disorder; BEAQ, Brief Experiential Avoidance Questionnaire; vs., versus; CRP, C-reactive protein; 11D ASC, 11 dimension altered states of consciousness questionnaire; M scale, Mysticism Scale; BSI, Brief Symptom Inventory; MQOL, The McGill Quality of Life Questionnaire; LAP-R, The Life Attitude Profile-Revised; LOT-R, Life Orientation Test-Revised; PIL, Purpose in Life test; DTS, Death Transcendence Scale; PEQ, Persisting Effects Questionnaire; FACIT-Sp, Functional Assessment of Chronic Illness Therapy; SROS, Spiritual-Religious Outcome Scale; FMS, Faith Maturity Scale; EORTC-QLQ-30, European Cancer Quality of Life Questionnaire; FS, Flourishing Scale; WSAS, Work and Social Adjustment Scale; WEMWBS, Warwick-Edinburgh Mental Well-being Scale; SIDAS, Suicidal Ideation Attributes Scale; PRSexDQ, Psychotropic-Related Sexual Dysfunction Questionnaire; LEIS, Laukes Emotional Intensity Scale; EBI, Emotional Breakthrough Inventory; PTCS, Post-Treatment Changes Scale; RRS, Ruminative Response Scale; VAL, visual analog scale; dFC, dynamics of functional connectivity; STAR-C, Scale to Assess Therapeutic Relationship – Clinician version; STAR-P, Scale to Assess Therapeutic Relationship – Patient version; MGH-ATRQ, Massachusetts General Hospital Antidepressant Treatment History Questionnaire; MINI, Mini International Neuropsychiatric Interview; MSI-BPD, McLean Screening Instrument for Borderline Personality Disorder; ASRS, Adult Self-Report Scale; EQ-5D-3L, Euro QoL-5 dimension-3 level*.

An open-label feasibility study of psilocybin therapy (10 mg) then 7 days later 25 mg, of 12 people diagnosed with treatment-resistant depression (TRD) showed that 67% of participants had significantly reduced depression symptoms (measured by MADRS) at 1 week, with 40% of participants showing a sustained response at 3 months post-dose (6). Measures of anhedonia, which overlap with reward dysfunction (see below) and anxiety, which overlap with threat processing (as discussed above) also improved (Table 1). Furthermore, in some participants these antidepressant and anxiolytic effects were sustained at 6 month follow up (7).

A randomized, waiting list-controlled clinical trial, though still without a placebo control, confirmed the immediate and sustained antidepressant effects of psilocybin therapy in (non-treatment resistant) MDD (4). This study also comprised two psilocybin sessions but at higher doses (20 mg/70 kg and 30 mg/70 kg) than the previous study. This study showed that 16 participants (67%) at week 1 and 17 (71%) at week 4 had a clinically significant response (GRID-HAMD), whereas 14 participants (58%) at week 1 and 13 participants (54%) at week 4 were in remission (4). A phase 2, double-blind, randomized, controlled trial (*n* = 59) showed that psilocybin therapy was at least as effective as escitalopram in reducing depressive symptoms in MDD (5). Preliminary data from a phase 2b TRD trial (*n* = 233) demonstrated that psilocybin 25 mg resulted in a statistically significant treatment difference of −6.6 points on change from baseline in MADRS total scores compared to 1mg dose at week 3 (8) (Table 1). Whereas exploratory studies are underway to determine the safety and efficacy of psilocybin therapy in conjunction with SSRI's (102). Interestingly, a recent double-blind, placebo-controlled, cross-over study in 23 in healthy controls (HCs) who received 14 days of escitalopram or placebo prior to psilocybin (25 mg), suggested that escitalopram had minimal effects on subjective, pharmacokinetic, or physiological readouts (103).

It is established that the limbic system and specifically the amygdala (104, 105) are important transdiagnostic nodes in the therapeutic modulation of negative-positive valence systems. Hyper-reactivity of the amygdala is associated with negative processing/affectivity and an attentional bias to negative valenced information, which can occur across a range of stress related disorders, such as depression and various anxiety disorders (106–109). Increased access to information flow from the limbic system during psychedelic therapy is one of the mechanisms thought to underlie therapeutic change (13). In keeping with a recalibration of NVS and PVS responsivity, several studies in HCs have demonstrated attenuation of amygdala reactivity, associated with predilection toward positive compared to negative stimuli in the acute phase post-psilocybin (110–112). This effect may be sustained for up to 1 month (113). Overlapping effects have also been demonstrated for LSD in HCs, which impaired the recognition of sad and fearful faces (114) and reduced reactivity of the left amygdala and the right medial prefrontal cortex (mPFC) relative to placebo during the presentation of fearful faces (115). Very low dose LSD (13 mcg) decreased amygdala connectivity with the left and right postcentral gyrus and the superior temporal gyrus, and increased amygdala seed-based connectivity with the right angular gyrus, right middle frontal gyrus, and the cerebellum in 20 young HCs, though there were “weak and variable effects on mood” (116). While not investigating the amygdala, a recent pilot randomized trial in HCs, perhaps limited by a small sample size of 22, did not show acute or protracted alterations in the recognition of emotional facial expressions after a single dose of ayahuasca (117).

In contrast to the above studies in HCs, which generally show decreases in amygdala reactivity, an open label study of 19 antidepressant free TRD subjects, found increased amygdala responses to emotional faces 1 day after psilocybin (84). In the same cohort of TRD participants, decreased cerebral blood flow in the amygdala correlated with reduced depressive symptoms 1-day post-psilocybin (40). While the loss construct encompasses several transdiagnostic components, rumination and increased self-focus may be particularly important transdiagnostic psychedelic therapy targets. Rumination refers to recursive self-focused negative thinking and is a component of a variety of disorders across mood, anxiety, addiction, and some personality disorders (118–120). The aforementioned TRD study showed that decreased ventromedial prefrontal cortex-right amygdala functional connectivity during face processing was associated with reduced ruminative thinking at 1 week (85).

The corticolimbic system and the immuno-endocrine system are intrinsically linked. However, at this point limited conclusions can be drawn about the loss construct and immuno-endocrine mechanisms. An 8-week social isolation model in juvenile marmosets, resulted in decreased fecal cortisol levels in both ayahuasca and saline treated groups, though in the male animals, ayahuasca reduced scratching behavior and increased feeding (121). In humans, a single dose of ayahuasca acutely increased salivary cortisol levels in both TRD patients and in HCs in a parallel arm, randomized double-blinded placebo-controlled trial (92). Before ayahuasca the TRD group had a blunted salivary cortisol awakening response and hypocortisolemia compared to HCs, though 48 h after ayahuasca there were no differences in the cortisol awakening response or plasma cortisol levels between the groups (92). In the same cohort ayahuasca reduced C-reactive protein (CRP) levels in both TRD (which were higher at baseline) and HCs compared to placebo, though this may be related to the increases in cortisol (89, 93). The TRD group treated with ayahuasca showed a significant correlation between larger reductions of CRP and lower depressive symptoms 48 h after ayahuasca (93). However, there were no significant changes in IL-6 levels (93).

A non-controlled study of 11 HCs, that analyzed salivary cortisol and immune markers 30 min before after 90 min after inhaled 5-methoxy-N,N-dimethyltryptamine (5-MeO-DMT) found a significant increase in cortisol levels and decrease in IL-6 concentrations, whereas there were no changes in CRP and IL-1β (122). Although this was an exploratory study, neither the cortisol nor the immune markers correlated with subjective experiences (122). The precise impact of psychedelic induced acute cortisol activation and whether this is a therapeutic component is not fully clear, nor is the predictive implications of baseline cortisol or hormonal levels on the response to psychedelic therapy or on sustained effects. Similarly, the clinical relevance of the immune-modulatory effects is not yet clear.

### Fear and Threat Systems

When threat systems become excessively or repeated activated, which then exceeds an organism's ability to meet the demands (allostatic overload), psychopathology may ensue (123, 124). Psychedelics modulate acute and sustained fear/threat responses. A study in mice injected with low doses of psilocybin resulted in extinguished cued fear conditioning significantly more rapidly than high-dose psilocybin or saline-treated mice (125). A previous study in rats showed that N,N-DMT initially resulted in anxiogenic responses, but the long-lasting effects tended to reduce anxiety by facilitating the extinction of cued fear memory (126). Similarly, chronic, intermittent, low doses of DMT produced enhanced fear extinction learning without impacting working memory or social interaction and exhibited an antidepressant-like effect in the forced swim test (FST) in rats (127).

A recent study in male mice using the relatively selective 5-HT2A/2C receptor agonist DOI (1-(2,5-Dimethoxy-4-iodophenyl)-2-aminopropane) showed that it accelerated fear extinction, reduced immobility time in the FST, increased the density of transitional dendritic spines in the frontal cortex, and for the first time showed epigenetic changes in enhancer regions of genes involved in synaptic assembly which lasted for 7 days, in conjunction with more transient transcriptomic changes (128). The clinical relevance of putative epigenetic changes in humans are not yet clear (129).

From the neuroendocrine mechanistic perspective, a study of psilocybin treatment in male mice, showed that psilocybin acutely increased plasma corticosterone and anxiety like behaviors in the open field test (OFT) (130). The acute anxiogenic effects correlated with the post-acute anxiolytic effects and chronic corticosterone administration suppressed the psilocybin induced acute corticosterone and behavioral changes (130). The authors postulated that psilocybin may act as an initial stressor that provides resilience to subsequent stress (130). Indeed, this transient acute anxiety and subsequent attenuation of anxiety can occur in some individuals who undergo psychedelic therapy. It is important to note that not all pre-clinical studies are consistent, in part due to strain and model effects. The aforementioned study did not find significant changes in the sucrose preference test or the FST following psilocybin in C57BL/6J male mice (130), echoing a previous study which did not show effects of psilocin or psilocybin on the FST or in the OFT in Flinders Sensitive Line rats (131).

Another rodent study comparing psilocybin to the N-methyl-D-aspartate receptor antagonist—ketamine—showed that rats that received psilocybin and 5-min weekly arena exposure for the first 3 weeks exhibited significantly less anxiety-like behavior in the elevated plus-maze (EPM) compared to controls, whereas rats that received the ketamine and weekly arena exposure did not display a significant decrease in anxiety in the EPM (132). Rats that received psilocybin or ketamine and no arena exposure did not display a significant decrease in anxiety in the EPM (132). The authors postulated that psilocybin facilitates a period of “behavioral flexibility” in which exploration of a non-home-cage environment reduces their anxiety during future exploration of a novel environment (132). In the same study, psilocybin decreased immobility in the FST for up to 5 weeks after administration compared to control rats, whereas ketamine injected rats displayed decreased immobility up to 2 weeks, suggesting a longer lasting therapeutic effect of psilocybin over ketamine (132). It will be intriguing to see if clinical trials comparing psilocybin to ketamine reproduce the putative longer lasting therapeutic effect of psilocybin (NCT03380442).

In humans, dysregulated fear and threat responses underlie a range of psychiatric disorders and psychedelic therapy may revise dysregulated or maladaptive fear/threat responses. A review of 20 human studies of psychedelics in ICD-10 anxiety disorders from 1940 to 2000, albeit of sub-optimal methodological rigor (e.g., lack of control groups, blinding and standardization), indicated improvements in anxiety levels (133). The subsequent clinical trials in people diagnosed with cancer (80–82, 134) and the studies in depression (4, 6) also suggest anxiolytic effects of psychedelic therapy.

One of the notable conditions associated with dysregulated fear conditioning (and avoidance of conditioned contextual cues), together with emotional regulation, and dysfunctional neural activity in cortico-amygdala circuits, involving exaggerated amygdala and attenuated mPFC activity, is Post-Traumatic Stress Disorder (PTSD) (109, 135–139). Other anxiety disorders share overlapping neurobiological pathways linked to fear/threat circuitry and attentional bias of negative valenced information, though there is variability in the fear evoking stimuli (106, 140, 141).

While PTSD overlaps with other conditions in the domains of hypervigilance, avoidance and altered emotional valance, the vivid re-experiencing of the trauma is perhaps a point of divergence from many other conditions. Memory reconsolidation dysregulation is a cardinal clinical feature of PTSD and memories can be strengthened or weakened according to new experiences. Classical psychedelics have the capacity to acutely enhance the vividness and recall of autobiographical memories (142) which in the context of psychedelic therapy requires great care and attention. These autobiographical memories are highly influenced by environmental inputs such as music (143), which is linked to increased parahippocampal cortex-visual cortex enhanced visual imagery, including imagery of an autobiographical nature (144). In terms of therapeutic utility, it is noteworthy that psilocybin leads to more vivid and visual recollections, associated with enhanced activation of visual and sensory cortical regions after viewing positive autobiographical memory cues (145). In terms of advancing the mechanistic understanding, undoubtedly future preclinical studies will delve into the impact of psychedelics on memory engram storage and retrieval (146, 147).

It is not known whether psychedelic therapy has the potential to augment therapies, such as cognitive processing therapy or prolonged exposure therapy in PTSD or indeed in any other anxiety disorder. However, there are preliminary indicators that psychedelic therapy may be useful in PTSD (148, 149). A retrospective, self-report survey of Veterans 30 days before and 30 days after participation in a psychedelic clinical program utilizing ibogaine and 5-MeO-DMT reported significant reductions in symptoms of PTSD, depression, anxiety, suicidal ideation and cognitive impairment (148). Increases in psychological flexibility (discussed below) were associated with the improvements in self-reported PTSD symptoms, depression, and anxiety (148). It will be interesting to ascertain whether the same psychedelic therapy induced modulation of cortico-limbic circuits (as discussed in section Loss construct above) will underpin therapeutic changes in PTSD and other anxiety disorders. As with all these studies, future challenges include precisely disentangling the contribution of psychedelics from psychotherapy, with some suggesting that the only way to definitively achieve this would be *via* the rather challenging process of administering psychedelic compounds under general anesthesia or sleep (150).

Excessive fear/anxiety may lead to maladaptive patterns of avoidance. Some of the potential therapeutic subjective experiences induced by psychedelics involve the transition from experiential (151, 152) and emotional (88) avoidance to acceptance. Interestingly, attachment avoidance at baseline may be linked with psilocybin-related challenging experiences (153). Similarly, high neuroticism has been associated with unpleasant/anxious reactions in 3,4-ethylenedioxymethamphetamine (MDMA) therapy (154). This again highlights the vital importance of preparation sessions, particularly pertinent in those with marked threat sensitivity/anxiety.

### Frustrative Non-reward

The neural circuitry underling aggressive reactions (in the context of negative emotions) involve amygdala hyper-responsivity coupled with hypoactivity of prefrontal regions, which overlaps with threat processing circuitry (155, 156). The frustrative non-reward construct refers to “reactions elicited in response to withdrawal/prevention of reward, i.e., by the inability to obtain positive rewards following repeated or sustained efforts.” This could potentially be associated with some aspects of depression or aggression (157). Sensitivity to frustration, particularly in relation to interpersonal rejection and negative emotions focused on others (158) are components of emotionally unstable personality (disorder) (EUPD) (borderline personality disorder). It has been proposed that psychedelic therapy could assist with emotion regulation, mindfulness, and self-compassion in people with EUPD (159). There are tentative indicators of potential utility. For example, a non-controlled observational study of 45 HCs who participated in an ayahuasca session reported significant improvements in mindfulness capabilities and emotional regulation in the subgroup with borderline-personality traits (Table 1) (160). However, it is premature to draw any conclusions about the utility of psychedelic therapy in EUPD or other maladaptive personality traits/disorders (161).

In terms of other personality traits, data suggests that psychedelics may increase openness (44, 162–164). Moreover, higher baseline scores in the personality trait of absorption (focused attention) (45, 46) and openness may be useful predictors of a therapeutic psychedelic experience, reportedly linked to increases in brain entropy as measured by fMRI (and experiences of “ego-dissolution”) (165), though 5-HT2AR binding did not appear to correlate with variations in openness (166, 167), highlighting the individual variability in 5-HT2AR levels after psilocybin and the complex relationship with subjective changes.

### Modulation of Neuroplasticity as a Transdiagnostic Mechanism

In terms of RDoC, structural and functional neuroplasticity broadly falls under molecular and cellular units of analysis and probably applies, at least some degree, to all domains. The ability of psychedelics to rapidly rewire neural circuitry by engaging plasticity mechanisms has given rise to the term—“psychoplastogens” (168–173). While, it is generally accepted that the quality of the subjective experience, dependent on the optimization of set and setting (context) is a critical component of the therapeutic mechanism of action of psychedelic therapy (87, 174), some propose that the subjective effects may not be necessary to produce long-lasting changes in mood and behavior (171).

The classical psychedelics may share glutamatergic activity-dependent neuroplastic effects with ketamine (175) and on a longer timescale, with some types of conventional antidepressants (176). A study in rats utilized fluorescence microscopy and electrophysiology techniques to show that changes in neuronal structure are accompanied by increased synapse number and function, and the structural changes in the PFC and increase in glutamate induced by serotonergic psychedelics appear to lead to BDNF secretion, neurotrophin receptor tyrosine kinase (TrkB) stimulation, and ultimately mammalian target of rapamycin (mTOR) activation (177). Furthermore, both LSD and ketamine activated cortical neuron growth mechanisms after <1 h, an effect which lasted for several days (178) and could be divided into an initial stimulation phase requiring TrkB activation and a growth period involving sustained mTOR and AMPA receptor activation (178).

In mice, a single dose of psilocybin resulted in a 10% increase in spine size and density in the medial frontal cortex, which occurred within 24 h and persisted for 1 month (179). In pigs, a single dose of psilocybin compared to saline resulted in 4% higher levels of hippocampal synaptic vesicle protein 2A (SV2A) and lowered hippocampal and PFC 5-HT2AR density (180). Seven days post-psilocybin, there was still significantly higher SV2A density in the hippocampus and the PFC, whereas there were no longer any differences in 5-HT2AR density (180). Previous studies showed psychedelics increase early response genes in the PFC (181, 182) and this was further confirmed by a rapid dose dependent preferential modulation of plasticity-related genes in the PFC compared to the hippocampus in rats (183).

A recent pre-clinical study compared ketamine to Tabernanthalog (TBG)—a water-soluble, non-hallucinogenic, non-toxic analog of ibogaine (184). Both TBG (50 mg/kg) and ketamine reduced immobility in mice in the FST, though the effects of ketamine were more durable and ketanserin blocked the effect of TBG (184). TBG promoted structural neural plasticity, produced antidepressant-like effects and in keeping with the transdiagnostic effects, also reduced alcohol and heroin-seeking behavior in rodents (184). A single lower dose of TBG (10 mg/kg) administered to mice after unpredictable mild stress, rescued deficits in anxiety like behavior and cognitive flexibility, associated with restoration of excitatory neuron dendritic spines (185), thus echoing the effects of ketamine (186), albeit *via* different primary pathways.

Notwithstanding the gap between animal and human studies in demonstrating molecular changes in plasticity, there are indicators of alignment with the pre-clinical data. For example, a magnetic resonance spectroscopy (MRS) imaging study in HCs showed psilocybin modulated glutamate levels in the medial PFC (187). In blood, one small preliminary clinical trial showed that 2 days after ayahuasca BDNF levels increased in both the TRD and the HC groups (90), whereas other studies in HCs showed that LSD increased blood BDNF levels (188, 189). However, BDNF levels did not increase in a recent randomized pilot study in 22 HCs after a single dose of ayahuasca (117).

## Modulation of Positive Valence Systems

PVS are primarily responsible for responses to positive motivational situations or contexts, such as reward seeking, consummatory behavior, and reward/habit learning.

### Reward System

Reward-pathway dysfunction is associated with a range of disorders (190, 191), including but not limited to mood (192, 193), anxiety (194, 195), addiction disorders (196, 197) and eating disorders (198, 199). Psychedelic therapy induced attenuation of maladaptive reward signaling, or a recalibration of reward/fear systems (PVS/NVS) may be useful targets across the various disorders. Psychedelics may alter maladaptive signaling in the mesolimbic reward circuitry, either indirectly *via* 5-HT signaling in the case of psilocybin or directly *via* activation of dopamine receptors (D1 and D2) like LSD (200, 201). A microdialysis study in awake rats found that intraperitoneal administration of psilocin significantly increased extracellular dopamine but not serotonin in the nucleus accumbens, increased serotonin and decreased dopamine in the mPFC, but neither were altered in the ventral tegmental area (202). An electrophysiological study in male mice showed that LSD altered neuronal activity in both the reticular and mediodorsal thalamus, partially mediated by the D2 receptor (34). Another recent study in chronically stressed male mice suggested that 5-HT2A independent mechanisms may be of importance in psilocybin induced anti-hedonic responses and associated cortico-mesolimbic reward circuit modulation (203).

The functional interaction between 5-HT and dopamine systems across molecular and neural networks was further expounded by a recent study in mice showing psilocybin increased FC between 5-HT-associated networks and resting-state networks of the murine DMN, thalamus, and midbrain, whereas it decreased FC within dopamine-associated striatal networks (204). It should be noted that this contrasts with the majority of human studies in HCs (as discussed below) that report acute decreases in DMN FC, thus highlighting the challenges of translation (32, 205–208).

In healthy humans, a structural MRI study showed a positive correlation between psilocybin induced feelings of unity, bliss, spiritual experience, and insightfulness subscales of the 5-Dimensional Altered States of Consciousness Rating Scale (5D-ASC) and right hemisphere rostral anterior cingulate thickness in HCs after controlling for sex and age (43). Whereas, a double-blind placebo-controlled study of 38 healthy experienced mediators that received psilocybin, reported positive changes in appreciation for life, self-acceptance, quest for meaning/sense of purpose at 4 months post-psilocybin (209). A pooled sample of HCs (*n* = 110) who had received between 1 and 4 oral doses of psilocybin (45–315 μg/kg) from eight double-blind placebo-controlled experimental studies (1999–2008), reported that the majority of subjects described the experience as pleasurable, enriching, and non-threatening (210).

A Positron emission tomography (PET) study in healthy humans showed that psilocybin increased striatal dopamine concentrations, and this increase correlated with euphoria and depersonalization phenomena (211), whereas the mixed 5-HT2/D2 antagonist risperidone attenuated the effects of psilocybin (212). This again re-enforces the divergence between the potential therapeutic benefit of psychedelic therapy in some reward dysregulated conditions, like depression, anxiety, and addiction, while exacerbating conditions like psychosis spectrum and manic disorders.

#### Addiction

The multi-layered complexities underlying addiction disorders are not only limited to reward and habit dysregulation but may include other constructs such as impulsivity and compulsivity (213). Compared to other recreational substances, psychedelics exhibit minimal reinforcing effects and are among the least harmful, with minor physiological side effects (24, 214, 215). Furthermore, preliminary clinical studies indicate a therapeutic use in alcohol use disorder, and for smoking cessation (216, 217). An open label pilot study of oral psilocybin in one or two supervised sessions in addition to Motivational Enhancement Therapy reduced alcohol consumption, which was maintained at 36 weeks, in a group of 10 participants with alcohol dependence disorder (10, 218). Although the mechanisms have yet to be fully elucidated, changes in alcohol consumption were associated with what is described as the “mystical” quality of the psilocybin experience (10).

Consistent with this, a subsequent online survey (*n* = 343) of people with prior alcohol use disorder, reported that insight, mystical-type effects, and personal meaning of experiences, together with higher psychedelic dose, were associated with a greater reduction in alcohol consumption (219). However, the potential mediating influence of negative and positive valence system modulation should also be acknowledged. Interestingly, neither psilocybin nor LSD administered in a high dosage regimen or chronic microdosing regime had long-lasting effects on relapse-like drinking in an alcohol deprivation effect rat model (220). Only sub-chronic treatment with psilocybin produced a short-lasting anti-relapse effect (220). A recent study showed that psilocybin restored alcohol dependence–induced metabotropic glutamate receptor (mGluR2) down-regulation and reduced alcohol-seeking behavior in rats (221). Interestingly, in a rodent food reward model, low dose psilocybin and ketamine failed to positively affect motivation or attention, though subtle improvements in attention and impulsive behavior were noted in “low performing” rats (222).

A pilot study of psilocybin and cognitive-behavioral therapy in people with tobacco addiction reported that 12 of 15 participants (80%) showed 7-day point prevalence abstinence at 6-month follow-up (9). Smoking cessation outcomes were significantly correlated with measures of mystical experience, of whom 9 of the 15 participants (60%) met criteria for “complete” mystical experience, defined as a score of ≥60% on each of the following subscales: unity, transcendence of time and space, ineffability, sacredness, noetic quality, and positive mood (223). A follow up qualitative study of participants (*n* = 12) reported vivid insights into self-identity, together with experiences of interconnectedness, awe, and curiosity which persisted beyond the duration of acute dosing (224). Clinical trials across a range of addiction disorders are currently underway to determine whether these promising preliminary studies progress to clinical utility (**Table 3**).

#### Depression

Reward hyposensitivity and decreased approach motivation is related to anhedonia, a cardinal feature of the Depression (192, 225). There are several psychological constructs by which psychedelic therapy may re-ignite reward deficits in states of anhedonia, including potential experiences of awe, curiosity, (explorative search), novelty, intrinsic motivation, psychological insight, and enhanced meaning/purpose (226). Conversely, reward hypersensitivity and elevated approach motivation is related to a subgroup of hypo/manic symptoms characterized by excessive approach motivation and psychomotor hyperactivation in the context of bipolar disorder (192). This reward hypo-hypersensitivity divergence maps onto the contra-indication of psychedelic therapy in bipolar type 1 disorders (BPAD I) (226, 227) and caution will be required in the treatment of the depressive phase of BPAD II (228). We await with interest the results of an open label safety and efficacy psilocybin (25 mg) therapy study in depressed participants with BPAD II and the future integration of dimensional approaches, such as reward-related reactivity assessments (**Table 3**).

## Modulation of Arousal and Regulatory Systems

RDoC's Arousal/Regulatory Systems are responsible for generating activation of neural systems as appropriate for various contexts and providing appropriate homeostatic regulation of such systems as energy balance and sleep (74).

### Arousal

Arousal is a continuum of sensitivity of the organism to stimuli, both external and internal. Several interacting systems are involved in arousal regulation, including but not limited to, the sympathomedullary and the immuno-endocrine system, which act as mediators to alter neural circuitry and function, particularly in the corticolimbic system. Psychedelics are highly context sensitive, “non-specific amplifiers” (229) of internal and/or external signals (immediate environment), in part due to the effects of 5-HT2AR signaling (230, 231). Psychedelics acutely modulate the Autonomic Nervous System (ANS) (39), neuroendocrine (232), and immune systems (233).

Psychedelics activate the sympathetic nervous system, including blood pressure, heart rate, body temperature, and pupillary dilation, probably *via* 5-HT2A and/or α1-adrenergic receptor-mediated mechanisms (114, 234–236). A recent randomized, placebo-controlled crossover trial in 25 HCs using electrocardiographic recordings showed that LSD increased sympathetic activity, which was positively associated with a range of subjective effects, measured by 5D-ASC (39). However, it should be noted that similar correlations were found for the placebo condition. In contrast, ketanserin increased parasympathetic tone and negatively associated with the subjective effects of LSD (39).

As discussed above, psychedelics also acutely stimulate the neuroendocrine system. In a seminal randomized placebo-controlled study of healthy experienced psychedelic users, IV DMT acutely and dose dependently increased blood cortisol, corticotropin, and other hormones such as prolactin and growth hormone (and ß-endorphin) (237). By 5 h post-dose, all endocrine markers returned to baseline values (237, 238). A double-blind, placebo-controlled study showed high dose psilocybin (315 μg/kg) acutely increased plasma ACTH and cortisol (and prolactin and thyroid stimulating hormone) in HCs (239). LSD (200 μg) increased plasma concentrations of the cortisol, cortisone, corticosterone, and 11-dehydrocorticosterone compared with placebo in 16 HCs using a randomized, double-blind, placebo-controlled cross-over study design (240). Other studies have also shown acutely increased plasma levels of cortisol, prolactin, oxytocin, and epinephrine due to LSD administration (234).

Psychedelics modulate the immune system *via* 5-HT1, 5-HT2, and sigma-1 receptor activity (18, 233, 241–248). Altered immune system function, mainly characterized by chronic low-grade inflammation is associated with a range of psychiatric disorders (57, 249–251) and it remains an open question whether the potential anti-inflammatory activity of psychedelics will play a role in autoimmune disorders (252) or chronic pain (253, 254).

### Sleep-Wakefulness

Sleep interference is almost ubiquitous across psychiatric disorders (255). Psilocybin (0.26 mg/kg) increased REM sleep latency in a randomized, double-blind placebo controlled cross over study of 20 HCs (256). Psilocybin suppressed slow-wave activity in the first sleep cycle but did not affect NREM sleep, EEG power spectra in NREM or REM sleep across the whole night (256).

## Modulation of Social Processing Systems

RDoC broadly defines systems for social processes as mediating responses in interpersonal settings of various types, including perception and interpretation of others' actions (74). The biologically encoded time-lagged personal narrative is constantly under the influence of bidirectional information exchange processes with the wider socio-environmental system. The multifaceted neural circuitry and molecular signaling pathways underlying social cognition, under the influence of environmental cues, are of fundamental importance to social species (257–259). A complex intertwined relationship exists between social isolation, disconnectedness, perceived disconnection, and poor mental health (158, 260). Psychedelic compounds alter social cognitive processes (Table 2) and studies in rodents are beginning to elucidate the underlying mechanistic pathways. A study in male mice showed that repeated doses of LSD (30 μg/kg, daily for 7 days), but not a single dose, resulted in more time interacting with a stranger mouse in the direct social interaction test, associated with potentiation of mPFC excitatory transmission *via* 5-HT2A and AMPA receptors and *via* an increasing phosphorylation of the mTORC1 protein (269). Moreover, the inactivation of mPFC glutamate neurotransmission impaired social behavior and negated the prosocial effects of LSD (269). Another study suggested that psilocybin attenuated some of the sociability deficits in a prenatal valproic acid mouse model of autism (270).

**Table 2:** Systems for social processes.

**Condition/measures**	**Design**	**N, Age**	**Psychedelic/dose**	**Clinical/neurobiological outcomes**	**References**
**Health controls** 5D-ASC, EDI rs-FC MRS	Double-blind, placebo-controlled, parallel group	60 HCs 30 psilocybin, F12, age 22.73 (2.90) 30 placebo, F13, age 23.20 yrs (3.65)	Psilocybin (0.17 mg/kg)	Psilocybin associated with acutely elevated medial PFC glutamate, correlated with negatively experienced ego dissolution Lower glutamate levels in hippocampal glutamate correlated with positively experienced ego dissolution Significantly less co-activation under the psilocybin vs. placebo in visual networks, both subcomponents of the DMN (anterior and posterior) and the auditory network Widespread increases in between-network FC observed under psilocybin vs. placebo	(187)
**Health controls** MEQ30, 11D-ASC, EDI PET: 5-HT2AR agonist radioligand [11C]Cimbi-36 Psilocin plasma concentration	Participants blind to dose	8 HCs (3F) Mean age 33.0 ± 7.1 yrs	Psilocybin between 3 and 30 mg	Subjective intensity ratings positively correlated with neocortical 5-HT2AR occupancy and plasma psilocin levels Positive associations mean intensity ratings and MEQ30, global 11-D- ASC score, and EDI score, and intensity ratings correlated also with both occupancy and with psilocin levels	(261)
**Health controls** 5D-ASC, PEQ	Double-blind placebo controlled 5-day silent retreat	39 HCs (experienced meditators) (15F) 51.66 yrs (± 8.32)	Psilocybin 315 mcg/kg	Psilocybin associated with increased meditation depth and positively experienced ego-dissolution Alterations in the DMN network, particularly a decoupling of medial PFC and PCC associated with subjective ego dissolution At 4 months post-psilocybin; positive changes in appreciation for life, self-acceptance, quest for meaning/sense of purpose	(208, 209)
**Health controls** 5D-ASC PANAS Social interaction task Social Influence paradigm fMRI and eye tracking	Double blind, randomized, counterbalanced, crossover	24 HCs (6F) 25.42 yrs (3.69)	(1) Placebo + placebo (179 mg mannitol/1 mg aerosil, po) (2) Placebo + LSD; 100 mcg po) (3) Ketanserin (40mg po) + LSD (100mcg, po) Aesthetic judgment task	LSD decreased the response to participation in self-initiated compared with other-initiated social interaction in the posterior cingulate cortex (PCC) and the temporal gyrus, more precisely the angular gyrus LSD decreased the efficiency of establishing joint attention ketanserin blocked effects LSD increased social adaptation but only if the opinions of others were similar to the individual's own Increases were associated with increased activity in mPFC while participants received social feedback Ketanserin blocked effects	(262, 263)
**Health controls** FFMQ, EQ, SC 2 MRIs (24 h pre and 24 h post-dosing) ^1^H-MRspectroscopy and resting-state BOLD	Open-label uncontrolled	16 HCs (6F) 38.9 yrs (±7.8)	Ayahuasca 0.3 mg/mL DMT Equivalent to 0.64 mg DMT/kg for 70 kg person	Reductions in glutamate + glutamine, creatine, and N-acetylaspartate+N-acetylaspartylglutamate in the PCC Glutamate + glutamine reductions correlated with increases in the “non-judging” subscale of FFMQ Increased connectivity between the PCC and the ACC, and between the ACC and limbic structures in the right medial temporal lobe Increased ACC-medial temporal lobe connectivity correlated with increased scores on the SC questionnaire Post-acute neural changes predicted sustained elevations in non-judging 2 months later	(264)
**Health controls** VAS 2 fMRIs	Within-subjects, counterbalanced Placebo-controlled	15 HCs (2F) 32 yrs (±8.9)	(1) receiving saline injection (“placebo,” PCB-session), 12 min task-free fMRI scan, eyes closed (2) 2 mg psilocybin infusion (“psilocybin,” PSI-session), midway through 12 min fMRI	Psilocybin-induced ego-dissolution was associated with decreased FC between the medial temporal lobe and high-level cortical regions and with a “disintegration” of the salience network and reduced interhemispheric communication Individuals with lower diversity of executive network nodes were more likely to experience ego-dissolution under psilocybin	(265)
**Health controls** HRS, 5DASC, M-scale, MEQ30, SOCQ, FMS, PEQ, DSES, DTS, GQ-6 Spiritual practices questionnaire Brief RCOPE	Double-blind, randomized	75 HCs (25 each group) (45F) 42 yrs (range 22–69)	(1) 1 mg/70 kg on sessions 1 and 2) with moderate-level (“standard”) support for spiritual-practice (LD-SS) (2) 20 and 30 mg/70 kg on sessions 1 and 2, respectively) with standard support (HD-SS) (3) 20 and 30 mg/70 kg on sessions 1 and 2, with high support for spiritual practice (HD-HS)	High-dose psilocybin produced greater acute and persisting effects vs. low dose At 6 months, compared with LD-SS, both high-dose groups showed large significant positive changes on longitudinal measures of interpersonal closeness, gratitude, life meaning/purpose, forgiveness, death transcendence, daily spiritual experiences, religious faith and coping and community observer ratings	(266)
**Health controls** Interactive virtual ball-tossing game (Cyberball) MRI, MRS	Double-blind, randomized, counterbalanced, cross-over study	HCs (*n* = 21) 26.48 yrs (SD = 4.76), range 20–37 yrs (9F)	Psilocybin 0.215 mg/kg po	Reduced feeling of social exclusion Reduced neural response in the dACC and the middle frontal gyrus compared to placebo Reduced neural response in the dACC significantly correlated with psilocybin induced changes in self-processing and decreased aspartate (Asp) content	(267)
**Health controls** Multifaceted empathy test and the moral dilemma task	Double-blind, randomized, placebo, controlled, within-subject design with 2 sessions (separated by 10 days)	HCs (*n* = 32) (5F) 26.72 ± 5.34 yrs, range 20–38 yrs	Psilocybin 0.215 mg/kg po	Increased explicit and implicit emotional empathy, compared with placebo No effect on cognitive empathy nor moral decision-making	(268)
**Health controls** 5D-ASC, AMRS, ARCI multifaceted empathy test Face emotion recognition task social value orientation test Acoustic startle response measurement	Double-blind, randomized, placebo-controlled, crossover	40 HCs (20F) 28.6 ± 6.2 yrs; range 25–51 yrs)	LSD (200 μg po) in 16 HCs and 100 μg LSD in 24 HCs	Subjective closeness to others, openness, and trust increased by LSD, enhanced explicit and implicit emotional empathy and impaired the recognition of sad and fearful faces, enhanced the participants' desire to be with other people and increased their prosocial behavior	(114, 234)

*F, female; QIDS, Quick Inventory of Depressive Symptoms; SHAPS, Snaith-Hamilton Pleasure Scale; STAI, The State-Trait Anxiety Inventory (STAI) trait scale (STAI-T); POMS, Profile of Mood States; HAMA, Hamilton Anxiety Rating Scale; GRID-HAM-D; HADS, Hospital Anxiety and Depression Scale; POMS, Profile of Mood States; HAM-D, Hamilton Rating Scale for Depression; MADRS, Montgomery-Asberg Depression Rating Scale; BPRS, Brief Psychiatric Rating Scale; YMRS, Young Mania Rating Scale; BHS, Beck hopelessness scale; SPECT, single photon emission tomography; CADSS, Clinician Administered Dissociative States Scale; PFC, prefrontal cortex; MRS, Magnetic Resonance Spectroscopy; EDI, Ego Dissolution Inventory; PEQ, Persisting Effects Questionnaire; FFMQ, Five Facet Mindfulness Questionnaire; EQ, Experiences Questionnaire; SC, short version of the Self-Compassion questionnaire; ACC, anterior cingulate cortex; 5D-ASC, 5-Dimensional Altered States of Consciousness Rating Scale; 11D-ASC, 11-Dimensional Altered States of Consciousness Rating Scale; EDI, Ego Dissolution Inventory; FFMQ, Five Facet Mindfulness Questionnaire; EQ, Experiences Questionnaire; SC, Self-Compassion questionnaire; PEQ, Persisting Effects Questionnaire; LSD, lysergic acid diethylamide; VAS, visual analog scale; M-scale, Hood's Mysticism Scale; SOCQ, States of Consciousness Questionnaire; FMS, Faith Maturity Scale; PEQ, Persisting effects questionnaire; DSES, Daily Spiritual Experience Scale; DTS, Death Transcendence Scale; GQ-6, Gratitude Questionnaire; MEQ, Mystical Experience Questionnaire; dACC, dorsal anterior cingulate cortex; HRS, Hallucinogen Rating Scale; AMRS, Adjective Mood Rating Scale; ARCI, Addiction Research Center Inventory*.

### Affiliation and Attachment

Experiences of disconnection or exclusion are common across psychiatric disorders and can manifest as social withdrawal, apathy, and anhedonia (260). Using a paradigm designed to induce feelings of social exclusion, a double-blind, randomized, counterbalanced, cross-over study of healthy participants (*n* = 21) reported that psilocybin induced reduced feelings of social exclusion (267) (Table 2). A placebo-controlled, double-blind, random-order, crossover study conducted using LSD (100 μg) in 24 HCs and LSD (200 μg) in 16 HCs, enhanced the participants' desire to be with other people and increased their prosocial behavior on the Social Value Orientation test (114, 234). In addition to significant positive changes in gratitude, life meaning/purpose, forgiveness and death transcendence, a double-blind study comparing low and high dose psilocybin therapy in HCs reported sustained increases in experiences of interpersonal closeness at 6 month follow up, associated with mystical-type experiences (266). It is interesting to note that psychedelics can increase oxytocin plasma levels (234), though the therapeutic relevance is not yet clear.

In keeping with possible increases in openness (210) and connectedness (88, 271, 272), studies have shown that psychedelic use may be associated with increases in nature relatedness (273–275), pro-environmental behaviors (276) and more broadly experiences of personal meaning (81, 148, 209, 219, 277). Taken together, psychedelic therapy induced changes in social processing systems and specifically social reward processing and behavior and enhanced experiences of connectedness (88) has potential therapeutic implications not only for depressive, anxiety, addiction, some personality disorders, but perhaps for social deficits in subtypes of adult autism spectrum disorders.

### Perception and Understanding of Others

There are preliminary indictors that classical psychedelics may enhance certain types of empathy (Table 2). LSD (114, 234) and psilocybin (268) acutely increased explicit and implicit emotional empathy, using the multifaceted empathy test and moral dilemma task in HCs, compared to placebo (268). Psilocybin did not affect the ability to take another person's perspective or affect the understanding of another person's mental state (cognitive empathy), nor did it affect moral decision-making (268). Using an aesthetic judgment task involving social feedback, LSD increased social adaptation to group opinions that were relatively similar to the individuals own opinions, associated with 5-HT2A activation and increased activity of the mPFC (263). Comparisons of psychedelic therapy delivered in individual settings compared to group settings offers an intriguing avenue to further explore how social processing domains and constructs such as perception and understanding of others may be shaped by the context in which the therapy is delivered. Non-controlled group studies have suggested that shared experiences, including acute relational experiences of perceived togetherness, may facilitate enhanced perception and understanding of others (272, 278). Controlled transdiagnostic studies directly comparing group to individual psychedelic therapy could decipher the relative therapeutic contribution of a group setting either before, during or after psychedelic administration.

### Perception and Understanding of Self

Notwithstanding the challenges of disentangling self from self-as social agent, current thinking implicates altered self-processing as the primary mode of action of psychedelic therapy with downstream implications for social processing systems (33). However, elucidating the precise temporal dynamics of altered self and self-as social agent, whilst also considering the pervasive emotional background is challenging. Nonetheless, the experience of a transient attenuation of the demarcation between self and other/environment or “ego dissolution” appears to be a pivotal transdiagnostic therapeutic mechanism (Table 2). This is especially relevant for excessive self-referential processes, which often manifest with negative valence. For example, ruminative or obsessional thoughts, which are components across a range of disorders, such as depression, anxiety disorders, eating disorders, addiction disorders and some types of personality disorders.

In contrast to disorders of constrained “self-focus,” which may benefit from a “broader spectrum of thought patterns and emotions” induced by psychedelic therapy (13, 33, 279), psychosis spectrum disorders appear not to benefit. This may be due to baseline features which include aberrant stability between intrinsic and extrinsic self-processing networks (280), aberrant salience attribution (281) and a loosening of higher-level priors (13). Some of these experiences are attenuated by second generation antipsychotics (e.g., clozapine, olanzapine, quetiapine, and risperidone), which block 5-HT2A and dopamine receptors (282). A previous study in HCs showed that risperidone attenuated the effects of psilocybin (212).

The intensity of psilocybin induced subjective experiences, including ego dissolution are dose dependent and appear to correlate with cerebral 5-HT2ARs occupancy and plasma psilocin levels (261). While the molecular cascade initiated by 5-HT2AR activation and downstream cortical glutamate modulation (24, 177) are key neurobiological substrates of self-processing alterations, the full molecular pathways and how they map onto the self-concept have yet to be fully determined, and at least in this regard, only partial assistance can be derived from preclinical models. From the perspective of refining personalized-precision psychedelic therapy, a PET study in 16 HCs showed that lower neocortical 5-HT2AR binding before psilocybin was associated with longer peak effects, a more rapid decrease in subjective drug intensity effects and higher scores on the Mystical Experience Questionnaire (283).

An MRS study in HCs showed that psilocybin acutely elevated mPFC glutamate, which was associated with negatively experienced ego dissolution, whereas lower levels in hippocampal glutamate secondary to psilocybin, were associated with positively experienced ego dissolution (187). A previous MRS study of 16 HCs 1 day after consuming ayahuasca showed reductions in glutamate and glutamine in the posterior cingulate cortex (PCC), which correlated with increases in the “non-judging” subscale of the Five Facets Mindfulness Questionnaire (264). Similarly, one week after psilocybin therapy, glutamate and N-acetylaspartate concentrations were decreased in the Anterior Cingulate Cortex (ACC) in an open-label study of 24 patients with MDD (83). A double blind, randomized, counterbalanced, crossover study of 24 HCs utilizing MRI and eye tracking showed that LSD decreased the response to participation in self-initiated compared with other-initiated social interaction in the PCC and the temporal gyrus, more precisely the angular gyrus (262) (Table 2).

### Neural Circuitry

One of the higher-order brain networks modulated by psychedelics that has gained attention in recent years is the DMN, associated with a range of experiences and conditions (284), including but not limited to self-reflection and rumination (13, 120, 265, 285, 286) and meta-cognitive processes (287). Alterations in DMN rsFC have been demonstrated across a range of disorders. However, a clear and consistent DMN signature specific to any disorder has yet to emerge, underscoring the complexities of mapping correlates of subjective experiences, but also the limitations of biosignature exploration utilizing categorical diagnoses.

Psychedelics reliably alter DMN circuitry and studies in HCs reported decreases in rsFC within the DMN induced by psilocybin (205), LSD (32, 207) and ayahuasca (206). In fifteen HCs intravenous psilocybin resulted in a significant decrease in the positive coupling between the mPFC and PCC (205). LSD (75 μg) 100 min after IV administration decreased connectivity between the parahippocampus and retrosplenial cortex and correlated strongly with ratings of ego-dissolution and altered meaning in 20 HCs (207). Notwithstanding the differences between experienced users who may be more receptive to psychedelic therapy compared to people with mental health disorders, ayahuasca resulted in a significant decrease in activity through most parts of the DMN, including the PCC and the medial mPFC in a group of ten experienced users (206). A decoupling of the mPFC and PCC was associated with positively experienced ego dissolution in a psilocybin double-blind placebo controlled study of 38 healthy experienced mediators (208). Furthermore, the meditators in the psilocybin group reported increased meditation depth and positively experienced ego-dissolution, while at 4 months post-psilocybin they reported positive changes in appreciation for life, self-acceptance, quest for meaning and sense of purpose (209). Interestingly, alteration of the DMN is not limited to classical psychedelics. Oral administration of MDMA (125 mg) to 45 HCs in a randomized, placebo-controlled, double-blind, crossover design showed decreased connectivity within the DMN, two visual networks, and the sensorimotor network (288). Another recent placebo controlled study of 12 healthy males using vaporized salvinorin A, acutely attenuated the DMN during peak effects (first half of 20 min scan) (289), highlighting the overlap with classical psychedelics.

Unsurprisingly given the complex multi-modal nature of self-processing, a single neural correlate such as the DMN may not fully capture the complexities of the self-processing concept (33, 290). Psychedelics alter global brain connectivity, of which the DMN is but one. For example, increased global FC correlated with ego dissolution in an LSD study of 15 HCs (291) and more recently the subjective effects of LSD have been shown to be non-uniform in time, depending on the particular state of the brain at a given point in time (290, 292), with multi-modal imaging techniques (fMRI, diffusion MRI, PET) highlighting the importance of 5-HT2A receptors (27). Previous studies in HCs showed that psilocybin (2 mg) IV destabilized a frontoparietal subsystem (293), whereas IV LSD (75 μg) and IV psilocybin increased the fractal dimension of bold blood oxygen level dependent (BOLD) time-series from regions assigned to the dorsal-attention network (294). Furthermore, a recent rsFC fMRI study in 10 healthy volunteers showed that the executive control network was decreased at 1-week, which was associated with increased mindfulness at 3 months, but there were no other significant changes in other networks (295).

From a personalized point of view, a study suggested that baseline brain connectivity may be a useful predictive marker (41). This double-blind, placebo controlled, randomized, cross-over study of 23 HCs who received oral psilocybin (0.2 mg/kg) and underwent resting-state functional connectivity fMRI scans at three time points (41) showed that psilocybin reduced associative, and concurrently increased sensory brain-wide connectivity over time from administration to peak-effects (41). Furthermore, the participants who had the lowest values in hyper-connected areas and had the highest values in hypo-connected regions displayed the strongest psilocybin induced changes in global brain connectivity (41).

In contrast to the aforementioned psychedelic induced acute decreases in DMN integrity in HCs, an open-labeled study in TRD (*n* = 20) reported an increase in DMN rsFC 1-day post-psilocybin (40). The reduction of depressive symptoms at 5 weeks was predicted by high scores of acutely experienced pleasurable self-dissolution and by low scores for dread of ego dissolution (87). Furthermore, the increased ventromedial prefrontal cortex-bilateral inferior lateral parietal cortex rsFC, 1-day post-dose, predicted treatment response at 5 weeks post-dose (40). Data from this study (*n* = 16) (40) combined with the psilocybin therapy vs. escitalopram study (*n* = 43) indicated that psilocybin was associated with a global decrease in network modularity, indicative of enhanced flexibility (as high modularity scores indicate a greater degree of separation between brain networks) (42). This decrease in modularity was associated with improvements in depression scores at 6-weeks as measured by the Beck Depression inventory (42). In contrast, no changes in modularity were observed with escitalopram, suggesting a tentative biomarker of response to psilocybin therapy.

## Modulation of Cognitive Systems

The RDoC organizes cognitive systems into attention, working memory, perception, memory (declarative), language, and cognitive control constructs.

### Cognitive Control

Cognitive control refers to a “system that modulates the operation of other cognitive and emotional systems, in the service of goal-directed behavior, when prepotent modes of responding are not adequate to meet the demands of the current context. Additionally, control processes are engaged in the case of novel contexts, where appropriate responses need to be selected from among competing alternatives” (74). This collection of executive control processes include goal-selection, maintenance, updating, as well as response selection and inhibition denotes the ability to switch between different mental sets, tasks, or strategies and plays a vital role in an individual's ability to adapt to environmental changes (296). The underlying neural circuitry involves the default mode, salience, and executive networks, with 5-HT2ARs playing an important role (297–299).

Psychedelics transiently impair certain aspects of cognition in a dose-dependent manner (142, 300–302). For example, a study in HCs showed that LSD (100 μg) compared to placebo acutely impaired executive functions, cognitive flexibility, and working memory on the Intra/Extra-Dimensional shift task, and Spatial Working Memory task, but did not influence the quality of decision-making and risk taking on the Cambridge Gambling Task (302). Similarly, psilocybin decreased attentional tracking ability in HCs, which the authors speculated was due to the inability to inhibit distracting stimuli (303). More recently, re-treatment with ketanserin (40 mg) normalized all LSD-induced cognitive deficits (302). Psychedelic induced impairment of aspects of cognitive flexibility was also observed in a probabilistic reversal learning paradigm in 19 HCs who received IV LSD (75μg) or placebo at two sessions, two weeks apart (Kanen 2021). In this study LSD resulted in more perseverative responding, though the reward learning rate and to a lesser degree the punishment learning rate were enhanced (304).

The complex relationship between cognitive flexibility, neural flexibility, and emotion has recently been highlighted by an open-label study of 24 patients with MDD (83). This study showed that psilocybin therapy decreased perseverative errors in a set-shifting task but did not impact response inhibition, selective attention, or abstract reasoning (83). The improvements in selective aspects of cognitive flexibility did not correlate with improvements in depression. Unexpectedly, greater increases in neural flexibility as measured by dynamics of FC (dFC) between the ACC and PCC, and greater baseline dFC from the ACC were associated with less improvement in cognitive flexibility (83). The practical inferences for the precise-personalized psychedelic therapy paradigm are not fully clear.

A retrospective survey self-report survey of U.S. Veterans in a psychedelic clinical program, reported significant reductions in cognitive impairment as measured by the Medical Outcomes Study—Cognitive Functioning subscale (148). However, changes in the negative valence domain may have led to secondary subjective improvements in the self-reported cognitive domains in this study. Similarly, limited conclusions can be drawn from a non-controlled study in self-selected HCs showing improvements in Cognitive flexibility and the Wisconsin Picture Card Sorting Task 24 h after ayahuasca, which the authors acknowledge could be attributed to practice effects (305).

The acute impairment in some executive domains induced by psychedelic compounds is especially relevant to neurodevelopmental disorders such as schizophrenia, which notwithstanding the inter-individual variability are associated with deficits in cognitive flexibility (306). The further acute impairment in cognitive control induced by psychedelics may in part explain the detrimental negative effects of these substances in psychosis or in those with predispositions to psychosis. Indeed, LSD induced “cognitive bizarreness” associated with loss of self-boundaries and cognitive control as measured by the 5D-ASC in 25 HCs (307) and “mind-wandering” (308) may be counterproductive for those at risk of developing psychosis.

A recent study focused on the claustrum, a thin sheet of gray matter, embedded in the white matter of the cerebral hemispheres and situated between the putamen and the insular cortex, with a rich supply of 5-HT2A receptors and glutamatergic connectivity to the cerebral cortex. The claustrum is thought to be associated with cognitive task switching (309, 310) and salience processing (311), known to be dysfunctional in psychosis (312). Psilocybin acutely reduced claustrum activity and altered its connectivity with the DMN and frontoparietal task control network (FPTC) in a study involving 15 HCs, thus implicating this region as a potential mediator in psilocybin therapy (310).

#### Obsessive Compulsive Disorder

OCD, frequently comorbid with anxiety and depression, involves deficits in cognitive control, goal-directed planning habit, reward processing (313–315) and negative valence system dysregulation, including abnormal fear extinction (316). Rodent studies have shown that psilocybin reduced digging in the marble burying test—a surrogate for compulsive behavior (317, 318). However, a recent rodent study showed that blockade of 5-HT2A or 5-HT2CRs did not attenuate the effect of psilocybin on digging, suggesting that a different mechanism dominates this effect (318). A psilocybin therapy proof of concept study of antidepressant free people diagnosed with OCD (*n* = 9) that had failed to respond to at least one SSRI, reported a 23–100% decrease in the Yale-Brown Obsessive Compulsive Scale in the 24 h following ingestion (YBOCS) (15) (Table 1). Limited conclusions can be drawn from this study due to lack of a control group and lack of a clear dose-response relationship to changes in the YBOCS. Results from ongoing clinical trials in OCD will give a clearer picture and it will be interesting to parse potential therapeutic effects of psychedelic therapy according to cognitive control, and negative and positive valence processing systems (Table 3).

**Table 3:** Currently registered clinical trials with psychedelics: potential for future integration of outcomes with RDoC.

**Categorical diagnosis**	**Psychedelic, dose, therapy**	**Measures**	**Negative-valence system**	**Positive-valence system**	**Cognitive systems**	**Social processing systems**	**Arousal/regulatory systems**	**Sensorimotor systems**
**Alcohol Addiction**								
**Alcohol use disorder** phase 2, randomized, double blind, placebo controlled, parallel *n* = 60 NCT04141501	Psilocybin 25mg, po, once (3-and 6-mo follow-up) Mannitol	TLFB, MET fMRI; rsFC cue-reactivity & Autobiographic memory bloods; genome-wide epigenetic markers ethylglucuronid, AST, ALT, GGT, Cortisol blood cells differentiated into cortical neurons	Potential threat (anxiety) Sustained threat	Reward responsiveness: anticipation, initial response, satiation Reward learning: probabilistic and reinforcement learning, habit Reward valuation: ambiguity/risk, delay, effort	Attention working and Declarative memory Cognitive control: goal selection, updating, response selection; inhibition/suppression	Affiliation and attachment Perception and understanding of self and others	Circadian rhythms sleep and wakefulnessarousal	
**Alcohol dependence** phase 2 *n* = 180 NCT02061293	Psilocybin 25mg/70 kg po at week 4, 25-40 mg/70 kg po at week 8 Psilocybin 25-40mg/70 kg at 38 weeks Diphenhydramine 50mg po at week 4, 50-100mg po at week 8	PACS, AASE, Readiness rulers, TLFB, SIP Motivational Enhancement and Taking Action (META)						
**Alcohol use disorder** *n* = 10 Open label Phase 2 NCT04718792	Psilocybin 25mg po once Blood psilocin levels	11-DASC, MEQ, AWE-S, EDI, PACS, AASE, MAAS						
**Other Addiction**								
**Nicotine dependence** *n* = 80 40 psilocybin 40 nicotine patch NCT01943994	Psilocybin (30mg/70kg) 13-week CBT for smoking cessation	Subgroup; 50 (25 per group) MRI week 2 before Target Quit Date & week 5 (if abstinent 3^rd^ MRI at 3 months)urinary cotinine, Breath Carbon Monoxide (CO)	Potential threat (anxiety) Sustained threat	Reward responsiveness: anticipation, initial response, satiation Reward learning: Probabilistic and reinforcement learning, habit Reward valuation: ambiguity/risk, delay, effort	Attention Working and declarative memory Cognitive control: goal selection, updating, response selection; inhibition/suppression	Affiliation and attachment perception and understanding of self and others	Circadian rhythms sleep and wakefulness arousal	
**Cocaine use disorder** *n* = 40 phase 2 randomized pilot NCT02037126	Psilocybin 0.36 mg/kg po Diphenhydramine 100mg po	fMRI: DMN rsFC Glutamate-Glutamine (Glx)in the anterior cingulate cortex and hippocampus urine cocaine metabolites criminal involvement outcomes						
**Opioid use disorder** phase 1 open-label NCT04161066	Psilocybin two doses po 4 weeks apart augmentation buprenorphine/ naloxone, plus guided counselling	OCS, MEQ, TLFB, GSES, MLQ, BPI, GQ, COWS						
**Methamphetamine use disorder** *n* = 30 single blind, randomized, parallel phase 1 & 2 NCT04982796	Psilocybin twice (25mg & 30mg two weeks apart) plus 6-week psychotherapy during residential rehabilitation program	Self-report methamphetamine use and urine Stimulant Craving Questionnaire-Brief, BDI, SDS, GAD-7, Experiences in Close Relationships-Short form CRP, IL-6, TNF-a, IL-8, IL-10		Reward responsiveness Reward learning Reward valuation		Affiliation and attachment perception and understanding of self and others		Habit
**Eating Disorders**								
**Anorexia nervosa** open-label pilot phase 1 *n* = 18 NCT04052568	Four moderate to high doses psilocybin, 20mg at the first session, then remain at previous dose, or increase by 5mg up to a max 30mg	HADS, EDQLS, EDE-Q, ANSOCQ BMI	Acute threat (fear) Potential threat (anxiety) Sustained threat	Reward responsiveness: anticipation, initial response, satiation Reward learning: probabilistic and reinforcement learning, habit Reward valuation: ambiguity/risk, delay, effort	Attention working and declarative memory Cognitive control: goal selection, updating, response selection; inhibition/suppression	Perception: somatosensory and visual Perception and understanding of self & others	Circadian rhythms sleep and wakefulnessarousal	Sensorimotor dynamics Habit
**Anorexia nervosa** open label phase 2 *n* = 20 NCT04505189	3 doses of psilocybin, max 25mg po	RMQ, EDE, EDE-Q fMRI (2) EEG (up to 5)						
**Anorexia Nervosa** open label phase 2 *n* = 20 NCT04661514	Psilocybin 25mg po once	EDE, PASTAS, BISS, YBC-EDS-SRQ, EDI, EDE-QS, QIDS, CIA, ED-RR, 5D-ASC						
**Depression and Neurological Conditions**								
**Mild Cognitive Impairment** or **early Alzheimer's Disease** and clinical depression symptoms open-label, phase 1, *n* = 20 NCT04123314	Psilocybin (15mg/70 kg week 4 and 15 or 25mg/70kg week 6)	CSDD, QOL-AD	Loss Potential threat (anxiety) Sustained threat	Reward responsiveness: anticipation, initial response	Attention working and declarative memory Cognitive control Language	Affiliation and attachment Perception and understanding of self	Circadian rhythms sleep and wakefulness arousal	
**Depression and anxiety in Parkinson's Disease** *n* = 10 open-label single-arm pilot NCT04932434	Psilocybin 10mg if tolerated 25mg 2 weeks later	MADRS, HAM-A, PROMIS apathy & Positive Affect and Well-Being scales neuro-qol (depression & lower extremity function, cognitive function, fatigue, concern with death and dying, social roles and activities scales	Loss Potential threat (anxiety) Sustained threat	Reward responsiveness Reward learning Reward valuation	Attention Working and declarative memory Cognitive control Language	Affiliation and attachment perception and understanding of self	Circadian rhythms sleep and wakefulness arousal	Sensorimotor dynamics
**Depression and Alcohol Addiction**								
**MDD** with co-occurring **Alcohol use disorder** double-blind, placebo-controlled phase 2 *n* = 90 NCT04620759	Psilocybin 25mg po oncbrief Motivational Interviewing intervention	GRID-HAMD, TLFB, QIDS-SR, STAI blood GGT, carbohydrate deficient transferrin, AST/ALT ratio	Loss Potential threat (anxiety) Sustained threat	Reward responsiveness Reward learning		Affiliation and attachment perception and understanding of self	Circadian rhythms sleep and wakefulness arousal	
**Major Depressive Disorder (MDD)**								
**MDD** randomised, double-blind, active-placebo-controlled *n* = 60 NCT03866252	Treatment arm: 100μg LSD (first session) and 100 or 200μg LSD (second session) po control arm 25μg LSD (first session) and 25μg LSD (second session) po	IDS-SR/C, BDI, SCL-90, EAQ, EHS, JHS, TAS, VAS, SCQ, 5D-ASC, MS, HAQ-T/P, AMRS-C/P, NEO-FFI, Religiosity Scale (Z-Scale), PEQ sleep; actigraphy blood BDNF salivary cortisol awakening responses macrophage migration inhibitory factor and interleukin-1 beta fMRI; DTI, ASL	Loss Potential threat (anxiety) Sustained threat	Reward responsiveness		Affiliation and attachment perception and understanding of self	Circadian rhythms sleep and wakefulness arousal	
**MDD** *n* = 60 randomized, double blind, placebo controlled, parallel phase 2 NCT03715127	Psilocybin 0.215 mg/kg, po, once mannitol po (placebo)	BDI, MADRS, 5D-ASC fMRI						
**MDD** *n* = 80 randomized, double-blind, parallel phase 2 NCT03866174 NCT04353921	Psilocybin 25mg po once or niacin 100mg po (placebo)	MADRS, SDS web surveys & telephone interviews at months 2, 3, 4, 5 and 6, 8, 10, 12, 14, 16, 18, 20, 22 and 24						
**MDD** *n* = 18 placebo-controlled, blinded phase 1 NCT03554174	2 experimental sessions 4 weeks apart two of the following three: 1) placebo 2) psilocybin (0.1mg/kg) 3) psilocybin (0.3mg/kg)	GRID-HAM-D, QIDS-SR16 EEG: auditory Long-Term Potentiation (LTP) task Rey Auditory Verbal Learning Test (RAVLT) (modified computer version) affective go/no task						
**MDD** double-blind placebo-controlled design *n* = 60 NCT03380442	Psilocybin 25mg po once Comparator: single intranasal 125mg ketamine/saline	QIDS, HAMD, MADRS fMRI before and one week after drug (self-referential processing) blood peripheral gene expression and molecules						
**MDD** & Healthy phase 1 *n* = 6 NCT04711915	open label, non-randomized, crossover, fixed order; 0.1 mg/kg DMT IV 0.3 mg/kg DMT IV	ASC, VAS (anxiety, tolerability), reinforcing effects EEG HR, BP						
**MDD** & Healthy *n* = 68 NCT04673383	Double-blind, randomised, placebo-controlled N,N- DMT fumarate IV (SPL026)	Safety and tolerability data MADRS						
**Treatment Resistant Depression (TRD)**								
**TRD** open label *n* = 20 NCT04739865	Psilocybin 25mg po once as adjuvant to SSRI	MADRS, CGI	Loss Potential threat (anxiety) Sustained threat	Reward responsiveness		Affiliation and attachment perception and understanding of self	Circadian rhythms sleep and wakefulness arousal	
**TRD** *n* = 15 open-label phase 2 NCT04433858	Psilocybin 25mg po once	MADRS						
**TRD** *n* = 16 NCT04698603	Open label, non-randomized, 5-MeO-DMT (GH001), inhalation	Safety and tolerability HR, BP, RR, O2 (%), temp, bloods; biochemistry, hematology, urinalysis, ECG MADRS, BPRS, CADSS, C-SSRS, PVT, DSST						
**Bipolar Depression**								
**Type 2 Bipolar Disorder (BP-II) Depression** open-label, *n* = 12 phase 2 NCT04433845	Psilocybin 25mg po	MADRS	Loss Potential threat (anxiety) Sustained threat	Reward responsiveness Reward learning				
**Obsessive Compulsive and Related Disorders**								
**OCD** phase 1 *n* = 30 (15 each group) NCT03356483	Psilocybin 0.25mg/kg, po, once niacin 250mg	Y-BOCS, A-YBOCS, MADRS, BDI, OBQ-44, OCI-R, OC-TCDQ, STAI, Q-LESQ-SF, MEQ, BABS, COM-R, SMiLE, CEQ, 5D-ASC, PANAS-X, PEQ, NRS, PEBS, IDAQ, MBDS, IOS, EPQ, AUDIT, UFEC, DUDIT, SRNU, PSQI, URICA, CGI, SDS, LOT-R, PI-R fMRI: rsFC cortisol, CRP, ACTH, IL-4, IL-6, IL-10, IL-12, INF-gamma, TNF-alpha	Acute threat (fear) Potential threat (anxiety) Others; uncertainty intolerance	reward learning & responsiveness to reward hypervigilant to reward feedback and opt immediate relief (reduction of anxiety) habit	Cognitive control goal selection, updating, representation, and maintenanceresponse selection, inhibition, or suppression, performance monitoring	Affiliation and attachment perception and understanding of self	Circadian rhythms sleep and wakefulness arousal	Motor actions action planning and selection, initiation, inhibition and termination, execution sensorimotor dynamics, habit
**OCD** *n* =15 phase 1 NCT03300947	3 groups; psilocybin 100mcg/kg psilocybin 300mcg/kg lorazepam 1mg po, once weekly for 8 weeks	YBOCS, MADRSEEG; Error Related Negative Potential (ERN) fMRI: functional connectivity between the Caudate Nucleus (CN) and Orbital Frontal Cortex (OFC)						
**Body Dysmorphic Disorder** *n* = 12 open-label phase 2 NCT04656301	Psilocybin 25mg po once	BDD-YBOCS	Acute threat (fear) Potential threat (anxiety)		Cognitive control			Perception; somatosensory & visual
**Trauma/PTSD**								
**PTSD, chronic Depression, MS, HIV, and SARS-CoV-2- Long Haulers Syndrome** *n* = 30 non-randomized phase 1 NCT05042466	Psilocybin plant medicine microdosing 1gm to 1/5 gm every 2^nd^ day for 8 weeks	GAF, BAM, PTSD Checklist for DSM-5 (PCL-5)	Sustained threat Loss		Attention working and declarative memory Cognitive control	Affiliation and attachment perception and understanding of self	Circadian rhythms sleep and wakefulness arousal	
**PTSD** phase 2, multicentre, fixed-dose open label *n* = 20 COMP201	Psilocybin 25mg		Sustained threat Loss		Attention working and declarative memory Cognitive control	Affiliation and attachment perception and understanding of self	Circadian rhythms sleep and wakefulness arousal	
**Generalized Anxiety Disorder (GAD)**								
**GAD** (Psi-GAD-1) *n* = 72 randomised triple-blinded active-placebo-controlled ACTRN 12621001358831	Two dosing sessions 3 weeks apart dose 1: 25mg psilocybin dose 2: 25 or 30mg (if dose 1 exhibits limited acute subjective response) comparator: diphenhydramine 75mg (or 100mg)	HAM-A, GAD-7, QIDS-SR, Mini-SPIN, AG-D, PDSS-SR, SDS, PWI, UBCS, AUDIT, DUDIT, self-reported number of cigarettes smoked, AIM, IAM, FIM	Potential threat (anxiety) Sustained threat	Reward responsiveness Reward learning Reward valuation	Attention working and declarative memory Cognitive control	Affiliation and attachment perception and understanding of self	Circadian rhythms sleep and wakefulness arousal	
**Pain/Headaches**								
**Fibromyalgia** *n* = 30 double-blind, placebo- controlled phase 2 NCT05068791	Psilocybin 0.36 mg/kg po or dextromethorphan 2.6 mg/kg po	Self-reported pain severity, PGIC, BPI	Loss	Reward responsiveness	Cognitive control Perception: somatosensory	Affiliation and attachment perception and understanding of self	circadian rhythms sleep and wakefulness arousal	Sensorimotor dynamics
**Migraine Headache** **Post-Traumatic Headache** *n* = 24 placebo controlled, randomized, crossover phase 1 NCT03341689 NCT03806985	Psilocybin 0.0143 mg/kg po, psilocybin 0.143 mg/kg capsule placebo: microcrystalline cellulose capsule	Migraine headache days, frequency, duration, intensity of pain/photophobia/nausea/vomiting/ phonophobia, functional disability	Potential threat (anxiety)		Perception; somatosensory & visual		Circadian rhythms sleep and wakefulness arousal	
**Adult ADHD**								
**ADHD** phase 2a (MinMed, 2021)	LSD microdosing			Reward anticipation, delay, receipt	Cognitive control working memory verbal fluency, executive function			

*AST, aspartate aminotransferase; ALT, alanine aminotransferase; GGT, gamma-glutamyltransferase; PO, orally; CBT, cognitive behavioral therapy; rsFC, resting state functional; DMN, default mode network functional; BMI, Body mass index; MADRS, Montgomery-Asberg Depression Rating Scale; BPAD II, Type 2 bipolar affective disorder; Y-BOCS, Yale-Brown Obsessive; A-YBOCS, Acute Yale-Brown Obsessive-Compulsive Scale; BDD-YBOCS, Yale-Brown Obsessive Compulsive Scale Modified for Body Dysmorphic Disorder; BDI, Beck Depression Inventory; EAQ90, Symptom Check List; EAQ, Existential Concerns Questionnaire; IDS-SR, IDS-C, Inventory of Depressive Symptomatology (self-rated and clinician-rated); FFMQ, Five Facet Mindfulness Questionnaire; EHS, Elliot Humility Scale; 5D-ASC, Dimensions-Altered States of consciousness; GRID-HAM-D, GRID-Hamilton Depression Rating Scale; QIDS-SR16, Quick Inventory of Depressive Symptoms; HAMD, Hamilton Depression Rating Scale; HAM-A, Hamilton Anxiety Ratings Scale; WSAS, Work and Social Adjustment Scale; SDS, Sheehan Disability Scale; OBQ-44, Obsessive Beliefs Questionnaire; OCI-R, Obsessive-Compulsive Inventory-Revised; OC-TCDQ, Obsessive Compulsive Trait Core Dimensions Questionnaire; STAI, State-Trait Anxiety Inventory; Q-LESQ-SF, Quality of Life Enjoyment & Satisfaction Questionnaire; MEQ, Mystical Experience Questionnaire; BABS, The Brown Assessment of Beliefs Scale; COM-R, The Community Observer Ratings of Changes in Subjects' Behaviour and Attitudes; SMiLE, Schedule for Meaning in Life Evaluation; CEQ, Challenging Experience Questionnaire; 5D-ASC, 5-Dimension - Altered States of Consciousness; 11-DASC, 11-Dimensional Altered State of Consciousness scale; PANAS-X, Positive and Negative Affect Schedule Expanded Form; PEQ, The Persisting Effects Questionnaire; NRS, Nature Relatedness Scale; PEBS, Pro-Environmental Behavior Scale; IDAQ, Individual Differences in Anthropomorphism Questionnaire; MBDS, Mind-Body Dualism Scale; IOS, Inclusion of Others in Self Scale; EPQ, Ethical Positions Questionnaire; AUDIT, Alcohol Use Disorders Identification Test; UFEC, Utilization of Facility and Emergent Care; DUDIT, Drug Use Disorders Identification Test; SRNU, Self-reported Nicotine Use; PSQI, Pittsburgh Sleep Quality Index; URICA, University of Rhode Island Change Assessment; CGI, Clinical Global Impressions; SDS, Sheehan Disability Scale; LOT-R, Life OrientfMEation Test Revised; PI-R, Padua Inventory-Revised; EDI, Ego Dissolution Inventory; PACS, Penn Alcohol Craving Scale; AASE, Alcohol Abstinence Self-efficacy; MAAS, Mindful Attention Awareness Scale; AWE-S, Awe Experience Scale; TLFB, Time Line Follow Back; MET, Multifaceted Empathy Test; PACS, Penn Alcohol Craving Scale; AASE, Alcohol Abstinence Self-Efficacy Scale; SIP, Short inventory of problems; CBT, Cognitive behavioural therapy; OCS, Opioid Craving Scale; GSES, Generalized Self-Efficacy Scale; BPI, Brief Pain Inventory; TGQ, Gratitude Questionnaire; COWS, Clinical Opiate Withdrawal Scale; MLQ, Meaning in Life Questionnaire; GQ, Gratitude Questionnaire; HADS, Hospital Anxiety and Depression Scale; EDQL, Eating Disorder Quality of Life Scale; EDE-Q, Eating Disorder Examination Questionnaire; ANSOCQ, Anorexia Nervosa Stages of Change Questionnaire; RMQ, Readiness and Motivation Questionnaire; EDE, Eating Disorder Examination; EDE-Q, Eating Disorder Examination Questionnaire; EDE, Eating Disorder Examination; PASTAS, Physical Appearance State and Trait Anxiety Scale; BISS, Body Image State Scale; YBC-EDS-SRQ, Yale Brown Cornell Eating Disorder Scale; EDI, Eating Disorder Inventory; EDE-QS, Eating Disorder Examination Questionnaire Short Form; CIA, Clinical Impairment Assessment; ED-RR, Eating Disorder readiness to change and motivation for change; CSDD, Cornell Scale for Depression in Dementia; QOL-AD, Quality of Life Alzheimer's Disease; IDS-SR/C; Inventory of Depressive Symptomatology; self-rated and clinician-rated; EHS, Elliot Humility Scale; JHS, Jankowski Humility Scale; TAS, Tellegen Absorption Scale; VAS, The Visual Analog Scale; SCQ, States of Consciousness Questionnaire; MS, Mysticism Scale; HAQ-T/P, Helping Alliance Questionnaire (therapist version; patient version); AMRS-C/P, Adjective Mood Rating Scale; clinician version; patient version; NEO-FFI, NEO-Five-Factor-Inventory; PEQ, Persisting Effects Questionnaire; DTI, Diffusion Tensor Imaging; ASL, Arterial Spin Labeling; CGI, Clinical Global Impression; ADHD, Attention deficit hyperactivity disorder; CADSS, Clinician Administered Dissociative States Scale; C-SSRS, Columbia-Suicide Severity Rating Scale; PVT, Psychomotor Vigilance Test; DSST, Digit Symbol Substitution Test; CRP, C-Reactive Protein; IL-6, Interleukin; TNF, Tumor Necrosis Factor; PGIC, Patient Global Impression of Change; BPI, Brief Pain Inventory; PROMIS, Patient-Reported Outcomes Measurement Information System; Neuro-QoL, Quality of Life in Neurological Disorders; BAM, Brief Addiction Monitor; MS, multiple sclerosis; UBCS, Ultra Brief Checklist for Suicidality; GAD-7, Generalized Anxiety Disorder 7-item Scale; PWI, Personal Wellbeing Inventory; Mini-SPIN, Mini-Social Phobia Inventory; AG-D, Agoraphobia Dimensional Scale; PDSS-SR, Panic Disorder Severity Scale - Self Rated; AIM, Acceptability of Intervention Measure; IAM, Intervention Appropriateness Measure; FIM, Feasibility of Intervention Measure*.

#### Eating Disorders

Eating disorders also involve elements of altered cognitive control/reward processing (319, 320), together with aberrant fear/threat encoding processes or threat sensitivity associated with body/food/weight gain/body perception. Enhanced psychological flexibility induced by psychedelic therapy has been proposed as a potential therapeutic mechanism of psychedelic therapy in eating disorders (321). While a preliminary study suggested a benefit of psychedelic therapy in improving depression and well-being scores in people with a self-reported lifetime diagnosis of an eating disorder (16), we await results from ongoing clinical trials (Table 3) to determine whether psychedelic therapy will lead to clinically meaningful benefits in those with eating disorders (322). It is worth noting the possibility that psychedelic therapy may be of utility for disorders related to compulsive overeating, perhaps better categorized as food addiction.

#### Psychological Flexibility

The “psychological flexibility” concept lacks precise definition, but broadly refers to the ability to recognize and adapt to various situational demands and shift mind-sets/behavioral repertoires (323). It is associated with divergent thinking (DT), a spontaneous and free-flowing pattern where many solutions are possible, with the prospect of novel idea generation. Convergent thinking (CT), in contrast, focuses on the delivery of a single solution. Deficits in psychological flexibility underlie a broad spectrum of psychopathologies. Excessively constrained thought may occur in depression, PTSD/anxiety, OCD, addiction and eating disorders, whereas excessively variable thought may occur in ADHD or some personality disorders (324) and unconstrained thought may occur in psychosis (31). Psychological flexibility has been proposed as a potential transdiagnostic mediator of psychedelic therapy (148, 325, 326). However, the precise impact of psychedelics on psychological flexibility or on DT and CT are not fully clear. For example, a recent double blind, placebo-controlled study of sixty HCs, all of whom had previous psychedelic experiences, found that psilocybin (0.17 mg/kg) acutely decreased CT, which remained decreased for 7 days, whereas measures of DT including fluency and originality decreased, and scores of novelty increased compared to placebo, which were associated with alterations in the DMN (187, 327).

### Attention/Working Memory and Memory (Declarative)

Psychedelics acutely and dose dependently impair attention (328, 329), memory task performance (142, 300, 302) and spatial working memory (212). On the other hand, it appears that other domains such as the recall and vividness of autobiographical memory may be accentuated (142–145).

### Language and Perception

A computational analysis of semantic and non-semantic language in HCs who received IV LSD (75 μg) and placebo reported that LSD was associated with unconstrained speech (increased verbosity and a reduced lexicon) which was noted to be similar to speech changes during manic psychoses (330). Automated natural language processing methods (331, 332) or digital text analysis (333) may have the potential to improve prediction of psychosis outcomes and there are early indicators that quantitative descriptions of psychedelic experiences derived using Natural Language Processing may play a role in predicting therapeutic outcomes or trajectory in psychedelic therapy (47).

Psychedelics may induce visual imagery (334–336), distortions in the perception of time and space (337, 338) and synaesthesia (339, 340). Auditory and tactile perceptual changes occur less frequently but can occur at higher doses (210, 341). The implications of alterations in these systems for personalized psychedelic therapy are not fully clear, though it is interesting to note the recent proof of concept study showing a role for psilocybin therapy in migraine suppression (17), a condition known to be associated with aberrant connections from the somatosensory cortex to the frontal lobe (342).

Psychedelics over-engage primary sensory cortices and mostly encompasses visual hallucinations (often geometric) with preserved insight monitoring whereas hallucinations in psychosis, are mostly related to overactivation of associative networks, mainly include auditory hallucinations and poor reality monitoring (341). Electrophysiological correlates of IV DMT induced complex visionary experiences during “breakthrough” periods in 13 HCs were associated with a delta/theta rhythmicity (343). A further analysis of the same EEG data with eyes closed reported an EEG wave signal similar to those observed during eyes-open visual stimulation (344). The changes in resting state EEG, which included decreased spectral power in the alpha/beta bands, accompanied by widespread increases in signal diversity, were not specific to the visual system, but also correlated with somatic and metacognitive/affective domains (343, 344). Interestingly, a recent EEG study in freely moving rats showed some overlap with human studies, with a time-dependent global decrease and desynchronization of EEG activity, particularly in the frontal and sensorimotor cortex (345).

Similar to the previously discussed vulnerability to adverse effects of psychedelics in people with incoherent self-concept/aberrant salience in the context of psychosis spectrum disorder, baseline dysfunction in the some of the perceptual systems may increase the risk of adverse events in psychedelic therapy. For example, there is limited high-quality data on the rare condition—Hallucinogen Persisting Perception Disorder (HPPD) (346–348), which in most cases is due to a “subtle over-activation of predominantly neural visual pathways that worsens anxiety after ingestion of arousal-altering drugs, including non-hallucinogenic substances” (347). The authors note that a personal or family history of anxiety and pre-drug use complaints of tinnitus, eye floaters, and concentration problems may predict vulnerability for HPPD (347).

## Sensorimotor Systems

Sensorimotor systems are primarily responsible for the control and execution of motor behaviors, and their refinement during learning and development (74). The Sensorimotor Dynamics subconstruct: “processes involved in the specification or parameterization of an action plan and program based on integration of internal or external information, such as sensations and urges and modeling of body dynamics. This process is continuously and iteratively refined *via* sensory information and reward-reinforced information.”

The highly complex Functional neurological disorders (FNDs), previously known as conversion disorders, involve not only sensorimotor, but also salience, central executive, and limbic networks (349–351). There are no modern era clinical studies of psychedelic therapy in FNDs and systematic reviews of studies from several decades ago are not able to draw firm conclusions due to small numbers of low-quality studies, often lacking control groups and valid outcome measures (352, 353). It is also worth noting that LSD (100 μg) increased sensory-somatomotor brain-wide and thalamic connectivity in 24 HCs, while concurrently reducing associative networks (32). Using a Roving Somatosensory Oddball Task and simultaneous EEG/fMRI in 15 HCs, the same researcher showed that psilocybin (0.2 mg/kg) disrupted tactile prediction error processing in the mPFC, associated with increased salience attribution to non-salient stimuli (354). It remains an open question whether the complex and disrupted sensorimotor modeling of body dynamics and the accompanying emotional processing in conditions such as FNDs (or indeed eating disorders) can be therapeutically modulated by psychedelic therapy (Table 3).

### Psilocybiome—An Additional Unit of Analysis?

In keeping with an interconnected systems based psychiatry paradigm that conceptualizes the individual as a complex composite of interacting systems across all levels of organization, it has been proposed that the microbial ecosystem (microbiome) may serve as an additional transdiagnostic unit of analysis in the RDoC framework (355, 356). At the interface between the individual and the environment, the microbiome is intrinsically linked to human health and may play a contributory physiological role in some psychiatric disorders (357–359). This microbial signaling system communicates with the brain through the gut-brain axis *via* the immune system (360), tryptophan metabolism (361), the HPA axis (362), the vagus nerve (363) and by the production of microbial metabolites, such as short chain fatty acids (SCFA's) (364). The microbiota-gut-brain (MGB) signaling system operates throughout life but is particularly important during early development when it influences the development of the neural circuitry underlying social, cognitive, and emotional brain domains (365, 366). Preclinical research has revealed that neurotransmission, neurogenesis, myelination, dendrite formation and blood brain barrier organization are partially under the influence of this MGB axis signaling system (367–371). At the behavioral level, MGB axis signaling modulates cognitive function and patterns related to social interaction, locomotor activity and stress management (362, 372, 373). The gut microbiome also modulates psychotropic drug metabolism and absorption, which in turn modifies gut microbiota composition (374–376). Thus, the gut microbiome is an unconscious processing system that contributes to emotional, cognitive, and behavioral regulation (377). Acute and sustained psychedelic responses are influenced by bidirectional biofeedback information signals from the periphery and the environment. Consequently, the interaction of the classical psychedelics and the microbiome and mycobiome (fungal community) and associated signaling pathways, together with the potential mediating influence of the microbiome on the interaction between psychedelic therapy and acute and sustained dietary behavioral patterns may have implications for the optimization of precise-personalized-systems based psychedelic therapy (378).

### Personalized Psychedelic Therapy

The precise-personalized transdiagnostic paradigm is not without critics and major challenges. As yet, it has not delivered discernible translational benefits to patients (379). Regrettably, there are no psychobiological signatures to guide clinical practice, which still involves clinical assessment and trial and error treatment approaches (380). It is not yet clear whether a transdiagnostic paradigm will add translatable precision to clinical psychiatry, which comprises the severe end of the dimensions (381, 382). Some argue that the RDoC's utility may be limited for the most serious of mental disorders, including dementia, autism, schizophrenia, and bipolar disorder, and may be more useful for depression, anxiety disorders (including PTSD/OCD) and some personality disorders (383, 384).

However, the precise-personalized integrative neuroscience framework is at an early evolutionary stage (385) and the divergence between therapeutic utility for some disorders and exacerbation of others, indicates a role for the RDoC constructs and associated underlying units of analysis, to enhance the understanding and application of psychedelic therapy. While transdiagnostic treatments are not unique to psychedelic compounds, the potential for psychedelics to induce profound transient changes in emotion, thought and perception with marked inter-individual variation, together with the potential to exacerbate underlying pre-dispositions to psychosis and mania (30, 226, 227) compels a greater emphasis on a precise-personalized paradigm. Echoing the general lack of personalized precision in clinical psychiatry, comprehensive clinical assessments are the only available method to identify and exclude participants with disorders that may be exacerbated by psychedelic therapy.

Notwithstanding the reliance on clinical measures, currently available strategies to optimize therapeutic outcomes involve refinement of pharmacotherapy and psychotherapy schedules, though the precise ratio has yet to be determined. It appears that body weight adjusted dosing, albeit over a narrow dosing range of 20–30 mg, may have limited impact on the subjective effects of psilocybin (386) and it remains to be seen whether potential pharmacological modulators such as 5-HT2A receptor gene polymorphisms influence therapeutic response. Moreover, the precise interaction of other psychotropics (SSRI, SNRI, antipsychotics, and mood stabilizers) (387) and psychedelic therapy has yet to be determined. From the psychotherapeutic angle, a high-quality systematized foundation is a vital (388), though there is major scope for the advance of personalization/individualization in the context of an RDoC framework. It will also be interesting to consider the implications of psychedelic therapy for the Neuroscience-based Nomenclature project, developed to progress a more precise neuroscience based psychopharmacological nomenclature (389).

There are preliminary indicators that the advances in the mechanistic understanding of psychedelics may translate into more precise-personalized approaches (41, 42). As translational psychedelic science advances, a complete understanding of the molecular cascades and bidirectional information exchange processes between internal and environmental systems will require analysis across genome, transcriptome, proteome, metabolome, microbiome, epigenome, connectome, physiome and exposome (environmental) levels (390). Deciphering the precise interaction between these systems may advance treatment personalization algorithms, perhaps assisted by advances in technology, such as virtual reality (391, 392), smartphones (393) and biosensors/biofeedback (394). Yet, it should be noted that even if the whole endeavor reduces down to an elaborate set of multi-layered fluctuating ones and zeros or some superposition thereof, or special molecular configurations and information processing pathways yet to be discovered, it is the relationship between the complex configurations underlying our experiences and the empathetic sharing and compassionate understanding of those experiences with others and the environment that is the matter of meaning and the potential of psychedelic therapy.

## Conclusions and Perspectives

Psychedelic science and its translational corollary psychedelic therapy are evolving rapidly. Advances in the mechanistic understanding of the underlying pathways, which involve multiple interacting systems may also prompt the development of novel compounds lacking undesirable properties. Several large scale RCTs will determine whether psychedelic therapy translates into the psychiatric clinic for a range of non-psychotic spectrum disorders. Given the translatable transdiagnostic antidepressant, anxiolytic, and anti-addictive therapeutic potential of psychedelic therapy, deconstructing categorical psychiatric diagnoses according to dimensional systems and constructs that align with neurobiological systems may advance more targeted applications, with the possibility of optimizing therapeutic outcomes. As such, integration of the RDoC transdiagnostic dimensional framework with psychedelic therapy as it advances toward the psychiatric clinic has potential to progress an interconnected systems based precise-personalized psychedelic therapy paradigm and narrow the translational gap between neuroscience and psychiatry.

Further insights can be gained from clinical studies in progress with psychedelic therapy although the extent to which they have been designed with this in mind may hamper efforts at integration. Additionally, evolution of multimodal prediction estimation algorithms based on dimensional psychobiological signatures may optimize the delivery of psychedelic therapy and ultimately augment clinical assessments. Apart from the vitally important context (as broadly defined), exploratory studies have proposed baseline functional connectivity patterns and cingulate cortical thickness, autonomic nervous system activity, together with psychological factors as therapeutic predictors. Further unraveling the complex and dynamic molecular cascades and information processing pathways across all levels of analysis from micro to macro, within and between psychiatric disorders and how they converge on the acute and sustained therapeutic subjective trajectory may enhance a more complete systems level understanding of psychedelic therapy and is an important objective for translational neuroscience.

## Limitations

This is a narrative review which attempts to conceptualize psychedelic therapy in the context of an evolving RDoC framework and primarily focuses on the effects of psychedelics. The psychotherapy aspect as it relates to RDoC is underdeveloped. This review does not focus on a systematic analysis of the potential side-effects/risks of psychedelic therapy.

## Author Contributions

JK wrote the manuscript. CG, GC, JP, AH, CK, and VO'K edited the manuscript. All authors contributed to the article and approved the submitted version.

## Funding

VO'K was supported by the Health Research Board (HRB) through HRB Grant Code: 201651.12553 and the Meath Foundation, Tallaght University Hospital. VO'K was the Principal Investigator (PI) on the COMPASS trials (COMP001, 003, 004) in Ireland. JK is sub-PI on the COMPASS trials (COMP 001, 003, 004) in Ireland. GC was supported by the HRB through (HRA POR/2011/23 and HRA-POR-2-14-647) and supported by a NARSAD Young Investigator Grant from the Brain and Behavior Research Foundation (Grant Number 20771). CG was supported by grant funding from MQ: Transforming mental health (MQ16IP13), the Global Brain Health Institute (18GPA02), and Science Foundation Ireland (19/FFP/6418).

## Conflict of Interest

The authors declare that the research was conducted in the absence of any commercial or financial relationships that could be construed as a potential conflict of interest.

## Publisher's Note

All claims expressed in this article are solely those of the authors and do not necessarily represent those of their affiliated organizations, or those of the publisher, the editors and the reviewers. Any product that may be evaluated in this article, or claim that may be made by its manufacturer, is not guaranteed or endorsed by the publisher.
